# Miniature anti-metal UHF RFID tag antenna loaded with embedded open stubs for enhanced bandwidth

**DOI:** 10.1038/s41598-026-51170-1

**Published:** 2026-05-01

**Authors:** Muthukannan Murugesh, Muhammad Firdaus Akbar, Nor Azlin Ghazali

**Affiliations:** School of Electrical and Electronic Engineering, Universiti Sains Malaysia (USM), 14300 Nibong Tebal, Pulau Pinang Malaysia

**Keywords:** Engineering, Materials science, Physics

## Abstract

A miniature tag antenna, which has a geometrical dimension of 20 mm × 20 mm × 3.32 mm (0.061λ × 0.061λ × 0.010λ, where λ is the free-space wavelength at 915 MHz) has been proposed for metal-mountable UHF RFID applications. A single-layer folded patch antenna incorporating two open stubs is designed to provide high inductance for frequency tuning, resulting in an enhanced operational bandwidth of 855–942 MHz defined using read range *R* ≥ 4 m criterion, where the tag maintains a minimum read range of 4 m. The width of the stubs can be adjusted to fine-tune the tag resonant frequency. These two stubs are symmetrically positioned to generate a broadside radiation pattern above the metal surface, ensuring optimal performance. The proposed tag antenna can be easily constructed using flexible foam and polyimide substrates. The read range of the proposed tag antenna was characterized experimentally by placing it on a 20 cm × 20 cm metal plate. With 4 W effective isotropic radiated power (EIRP), the tag antenna can achieve a maximum measured read range of approximately 7.9 m at boresight. It should be noted that minor differences between simulated and measured results may arise due to practical implementation factors, including the use of a packaged RFID chip during fabrication compared with the bare-die impedance model used in simulations. A thorough analysis has been conducted to comprehend the resonance characteristics, and an equivalent circuit model has been developed for designing and optimizing the tag antenna. It is important to highlight that the resonant frequency of the proposed tag remains stable and slightly impacted by the presence of a metallic object behind it.

## Introduction

Radio Frequency Identification (RFID) technology has become progressively popular in recent years because of its versatility, with a large range of practical applications in different industries like supply chain^1^, retails and factories^2^, hybrid electronics^3^, wearable^4^ and medical applications^5^. Ultra-high frequency RFID tag antennas facilitate wireless communication without requiring a direct line of sight, making them perfect for tracking objects remotely. These antennas are extensively utilized across various fields, including agriculture, manufacturing, healthcare, and sensing applications^6^. RFID systems operate across several frequency bands, such as Low Frequency (LF), High Frequency (HF), and Ultra High Frequency (UHF). Unlike LF or HF RFID tags, which typically utilize small inductive loop antennas that can operate efficiently with compact physical dimensions, UHF RFID tags rely on radiating structures whose size is inherently governed by the free-space wavelength (typically around 33 cm at 900 MHz). As a result, UHF antennas generally require larger physical footprints to achieve proper impedance matching and efficient radiation. This becomes particularly challenging in on-metal applications, where the proximity to conductive surfaces disrupts the antenna’s current distribution and detunes its impedance. UHF RFID systems are particularly preferred due to their numerous benefits, such as faster data transfer rates, longer read ranges, and smaller form factors. A passive tag generally consists of an antenna and a chip. The chip holds information related to the product, which the antenna relays to the RFID reader. Passive tags operate by receiving energy from electromagnetic signals emitted by the reader, which powers the chip for communication. In UHF RFID technology, the tag cost stands as a pivotal consideration for its practical application. Label-type tags^7^, typically featuring planar antennas, offer a pathway to cost-effective mass production, whether through etching or printing techniques. Consequently, the cost of tags is minimized. However, achieving completely planar antennas for tags intended for metallic objects remains a challenge.

A significant obstacle to the widespread adoption of UHF RFID systems is the issue of antenna impedance detuning, which occurs due to the presence of nearby objects, especially those made of metallic materials or those with high permittivity^8^. Label-type dipoles are commonly employed due to their cost-effectiveness and ability to be printed on thin films^9^. Despite their versatility, these antennas face performance issues when placed near metallic surfaces due to the conducting surface boundary condition. Metallic surfaces cause incidental electromagnetic waves to reflect with a phase reversal, which significantly impacts the antenna’s operation. The proximity of such surfaces alters the antenna’s radiation pattern, input impedance, and resonant frequency. These effects depend on the size, shape, and distance of the metallic object from the antenna^10^. Overcoming the impact of tagged object materials is a key challenge in tag antenna design. Effective functionality across various materials necessitates a broad antenna bandwidth^11^ and a low-profile design. Typically, the antenna impedance is matched with the tag chip impedance to ensure optimal power transmission, thereby enhancing antenna performance. Two different methods in designing RFID platform-tolerant tags involve integrating a high permittivity substrate to mitigate surface effects or incorporating a conductive ground plane within the antenna structure^12^. Other approaches involve utilizing artificial magnetic conductor (AMC)^13^ or electromagnetic bandgap (EBG)^14^ structures in tag antenna designs because of their zero-reflection phase characteristic. Nevertheless, the complexity of these structures leads to higher manufacturing costs and restricts their practical applications. For example, a cylindrical RFID tag antenna has been reported to maintain stable performance on metallic surfaces through structural modification^15^. However, such non-planar configurations may restrict compact integration in practical applications. Therefore, the development of simple and compact planar antenna designs remains essential for efficient on-metal RFID systems. In^16^, an alternative approach is proposed for designing a reversible RFID tag capable of operating across both ETSI and FCC bands. Nevertheless, the associated structural complexity may limit its suitability for compact and low-profile implementations. Similarly, in^17^, a metal-mountable RFID tag is introduced that enables screw fixation by exploiting a central current null. While this improves mechanical integration, the design relies on specific structural configurations, which may constrain design flexibility. Furthermore, in^18^, a dual-band RFID tag antenna is developed using a stacked integration of a folded dipole and patch elements to enhance read reliability in metal-rich environments. Although the design effectively utilizes frequency diversity to mitigate multipath effects, it requires multilayer structures and precise integration of multiple radiating elements, leading to increased fabrication complexity and overall thickness.

Efforts to miniaturize UHF tag antennas for metal-mountable applications must carefully balance size reduction with the preservation of radiation efficiency, often involving structural modifications such as shorting stubs, embedded reactive elements, or folded geometries. However, such miniaturization strategies can lead to trade-offs in bandwidth and read range, making compact UHF tag design on metal surface is a complex and delicate task. Another major challenge in RFID tag antenna design lies in the conventional dipole structure, which typically resonates at approximately half the operating wavelength. This inherently leads to electrically large geometries that are unsuitable for compact tagging applications, especially when the tags must be mounted on metallic or space-constrained surfaces. To address this limitation, numerous miniaturization strategies have been proposed in the literature. These include techniques such as meandering the radiating arms to increase the effective current path without enlarging the physical footprint^19^, employing high-permittivity substrates to reduce the guided wavelength and thereby reduce the antenna size^20^, utilizing folded patch and dipolar structures incorporating shorting stubs to introduce inductive loading and lower the resonant frequency^21^, embedding reactive elements such as capacitive and inductive stubs to fine-tune impedance and resonance^22^, and incorporating notches or slots into the radiators to create additional reactive effects and extend the effective electrical length^23,24^. Our recent contributions to the development of miniaturized tag antennas for metal-mounted applications have explored several distinct design strategies. In^25^, a planar patch antenna incorporating substantial embedded serial capacitance was introduced, which effectively increased the electrical length and extended the achievable read range (40 mm × 40 mm × 3.38 mm). In^26^, a zeroth-order resonant (ZOR) serpentine patch antenna with closely overlapped features was proposed, where strong capacitive coupling enabled frequency tuning and geometric miniaturization within a compact footprint (50 mm × 50 mm × 3.38 mm). In^27^, a planar tag antenna employing concentric step-impedance rings in close proximity was presented, generating multiple resonant paths and achieving broad impedance matching (40 mm × 40 mm × 3.32 mm). Although these designs demonstrated substantial read ranges, improved radiation efficiency, and effective impedance matching with the RFID chip. However, the tag antennas in^25–27^ have employed different design techniques to achieve substantial read ranges, improved radiation efficiency, and effective impedance matching. However, these designs are not suitable for smaller metal objects due to their lack of compactness.

The novelties and contributions of the proposed tag antenna are now highlighted:

- (a) A compact single-layer folded-patch UHF RFID tag antenna is proposed for on-metal applications, achieving reduced size while maintaining wideband performance.
- (b) Dual embedded open stubs are introduced to enhance impedance matching and enable effective frequency tuning across the UHF band.
- (c) A comprehensive equivalent circuit model is developed to explain the antenna behavior and validate the design mechanism.
- (d) Extensive parametric and experimental analyses are conducted to demonstrate stable performance, including wide operational bandwidth and reliable read range on metallic surfaces.

Additionally, the proposed tag antenna is specifically designed for operation in the U.S. UHF RFID band (902–928 MHz), in compliance with FCC Title 47 CFR Part 15.247. It is intended for on-metal applications, where metal surfaces can cause signal distortion and impedance detuning. To address this, the antenna uses a folded patch structure with embedded open stubs and a ground plane to maintain stable performance. The design is matched to the UCODE-9^28^ chip impedance (13 – *j*191 Ω at 915 MHz) for efficient power transfer. All testing was carried out under a 4 W EIRP limit, following FCC power guidelines. Although the design is targeted for the FCC band, the antenna demonstrates a wide operational bandwidth from 855 to 942 MHz, achieving a minimum read range of 4 m across this entire span, with up to 8 m at resonance. This makes the design potentially adaptable for other regional standards, including ETSI and ARIB. The structure of this paper is outlined as follows: The following section discusses the antenna design analysis, detailing the tag antenna configuration and key design parameters, along with its equivalent circuit model. The next section covers the fabrication of the prototype and measured results. Finally, the last section provides the conclusion of the paper.

## Methods

### Design analysis

**Fig. 1:**
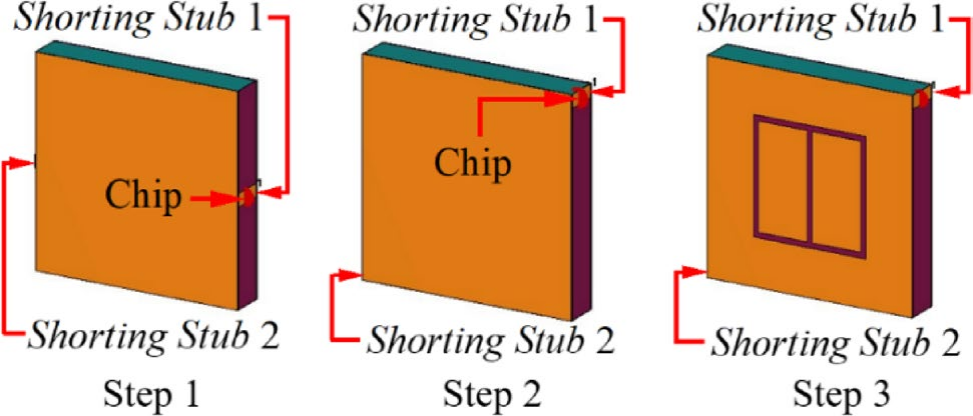
Design steps of the proposed on-metal UHF RFID tag antenna.

**Fig. 2:**
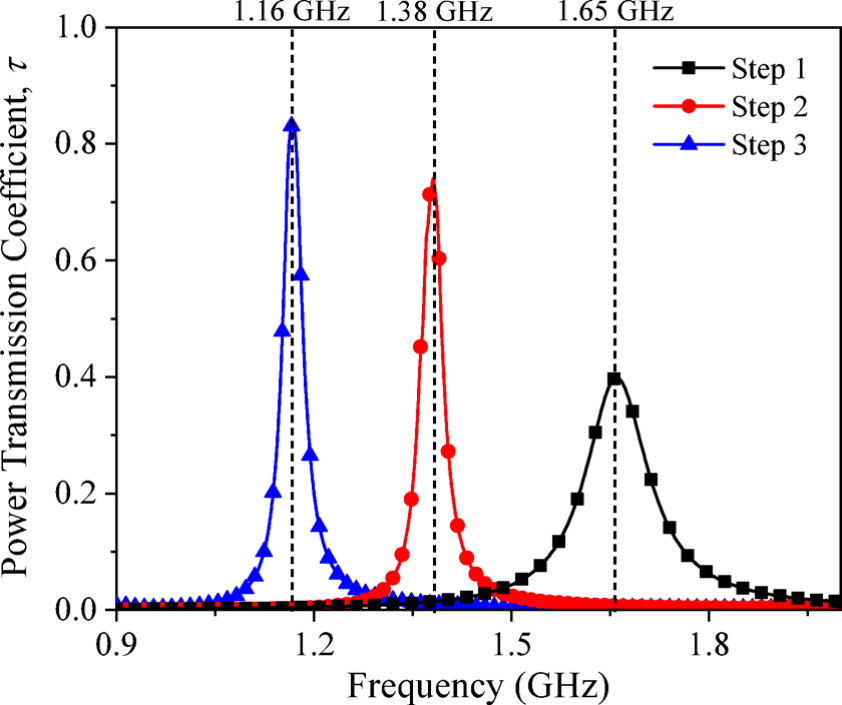
Power transmission coefficient (PTC) of the intermediate antenna configurations in Steps 1, 2, and 3.

**Fig. 3:**
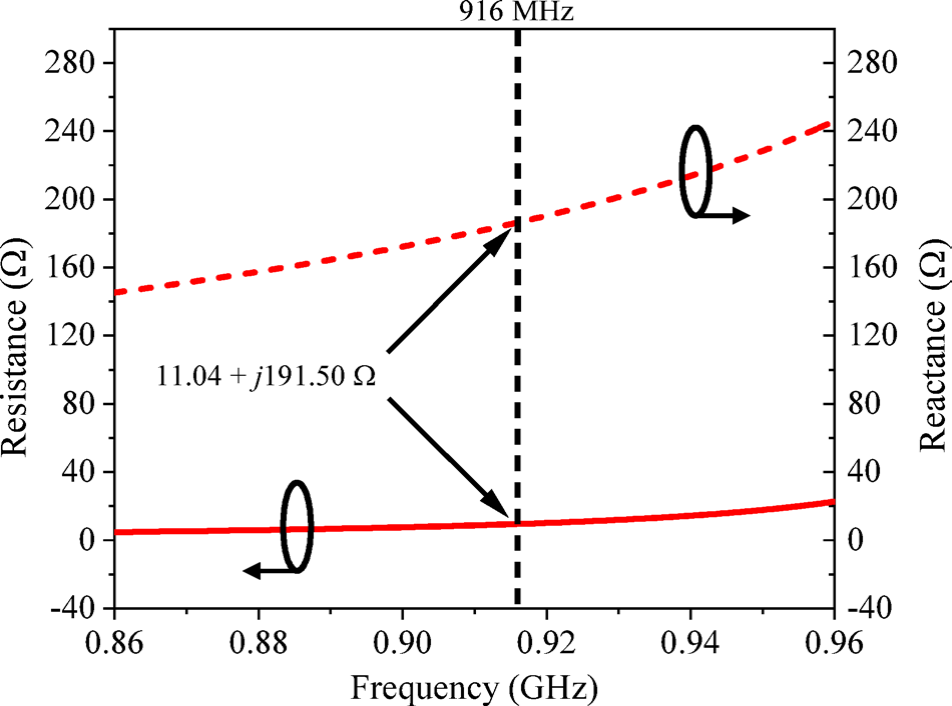
The proposed tag antenna’s simulated input impedance.

**Fig. 4:**
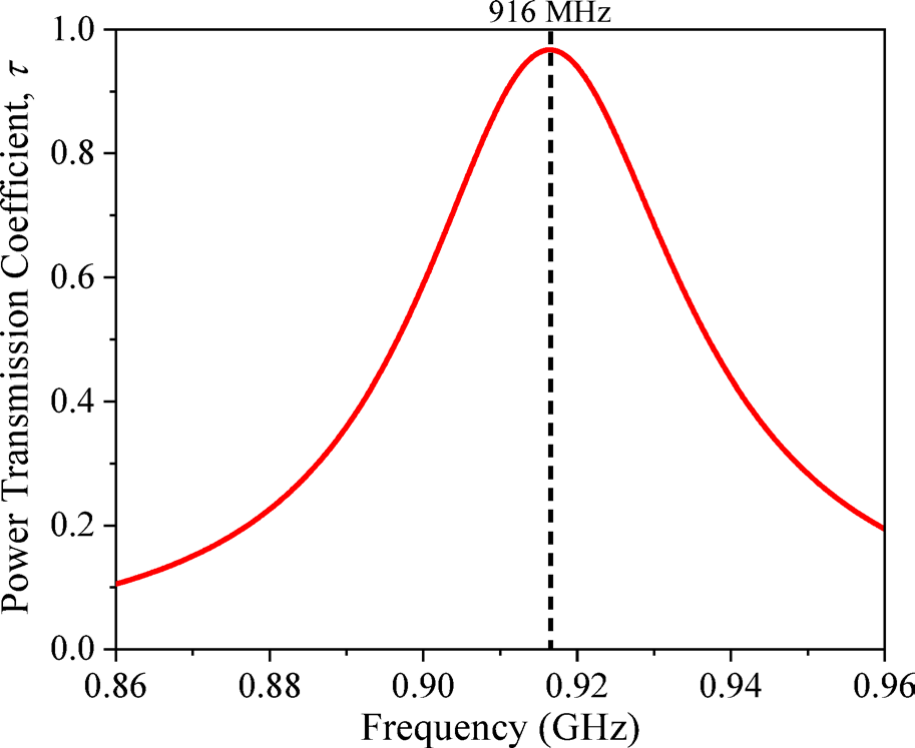
The proposed tag antenna’s simulated power transmission coefficient - PTC (*τ*).

The development of the antenna design is further explored by incorporating three intermediate tag antenna structures as shown in Fig. 1. Each of these structures is consistently positioned on a square metal plate with dimensions of 20 cm × 20 cm. The simulation begins by substituting each antenna terminal with a complex port impedance that represents the actual chip impedance. Initially, a basic single-layer square tag antenna (Step 1) is used, where the top layer is replaced with a complete metallic layer. Additionally, *Shorting Stub* 1 and *Shorting Stub* 2 are positioned in the middle of both sides. In this configuration, the tag’s resonant frequency is 1.65 GHz, which is significantly beyond the regulated UHF passband and the power transmission coefficient remains low at 40% as shown in Fig. 2. In Step 2, *Shorting Stubs* 1 and 2 are repositioned to the top right and bottom left corners to increase the electrical length of the tag antenna. This adjustment lowers the resonant frequency to 1.38 GHz and improves the power transmission coefficient to 70%. In Step 3, slots are introduced to generate a capacitive effect, further reducing the resonant frequency to 1.16 GHz. In this configuration, the power transmission coefficient improves to 82%. In the final configuration, open stubs are introduced, and the middle square patches are shorted to both sides to create a loading patch effect. The chip is relocated from the side *shorting stub* 1 to the top layer for easier chip bonding and port impedance measurements, which will be explained later. This optimized tag design achieves a resonant frequency of 916 MHz with a power transmission efficiency of 98%. Figure 3 illustrates the simulated impedance of the proposed tag antenna, as determined using CST Studio Suite simulation software. At the resonant frequency of 916 MHz, the simulated impedance is 11.04 + *j*191.50 Ω, closely achieving conjugate matching with the impedance of the UCODE-9 microchip. As shown in Fig. 4, a power transfer of nearly 98% is achieved, where good impedance matching is a crucial influencing factor.

## Tag antenna configuration

**Fig. 5:**
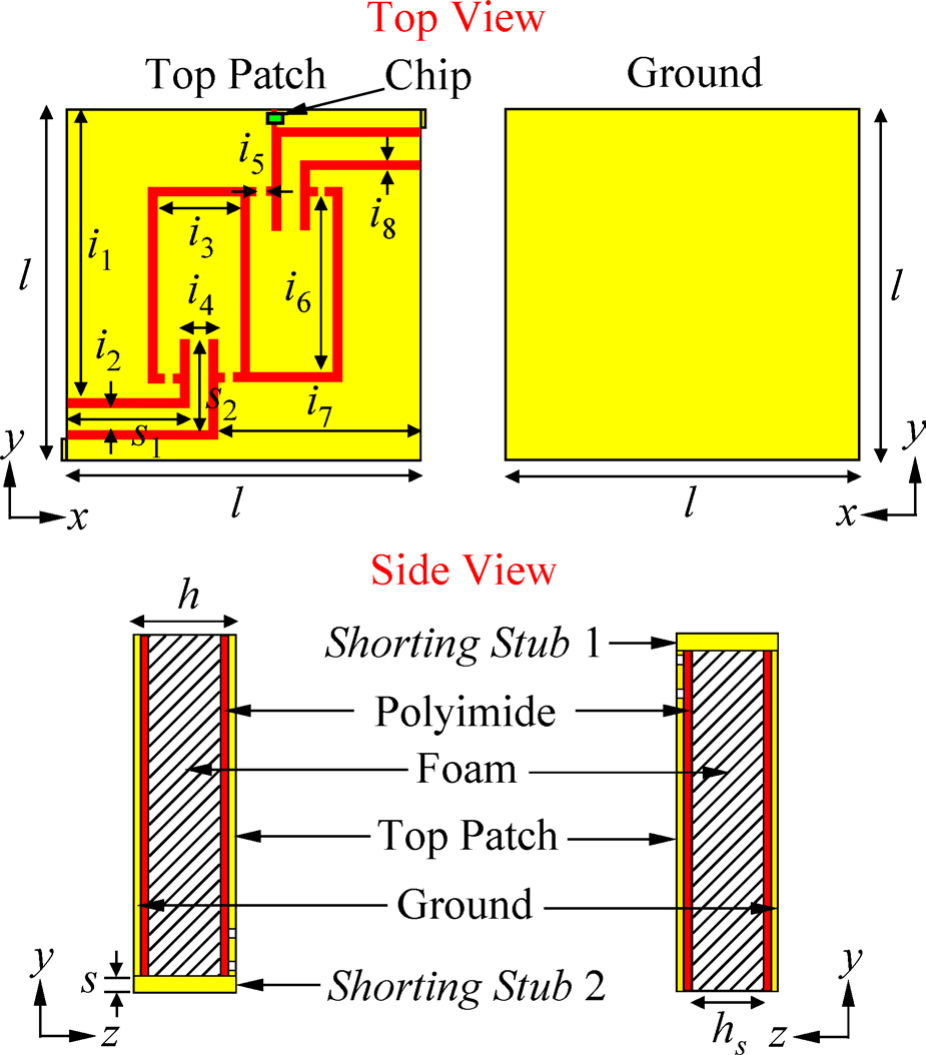
Structural configuration and geometry of the proposed on-metal UHF RFID tag antenna.

**Fig. 6:**
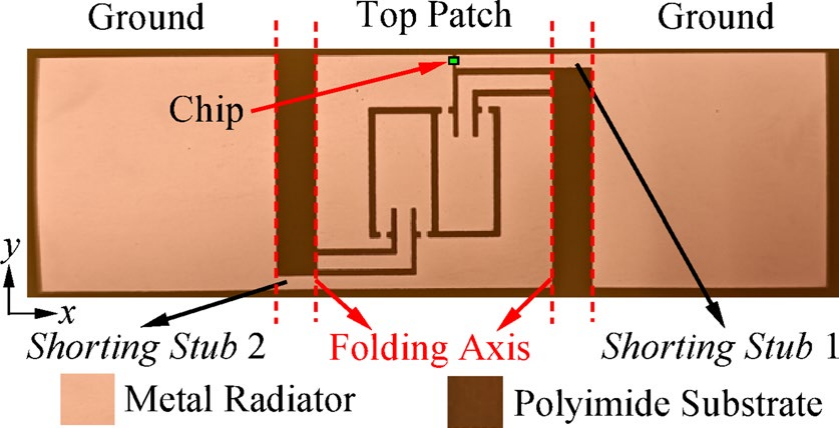
Layout of the single-sided RFID tag inlay for the proposed antenna.

The process begins with the antenna layout being transferred onto a thin copper sheet using a photoresist mask. After exposure to UV light, the excess copper is removed through chemical etching. The antenna inlay is created by etching away the undesired copper layer. The antenna design utilizes Rohacell 31 IG foam and polyimide (Kapton) film as the dielectric substrates. As shown in Figure. 5, the square-shaped foam substrate has a relative permittivity (*ε*_*r*_) of 1.03 and a loss tangent (tan *δ*) of 0.0001, specified by the manufacturer for frequencies near 900 MHz. It serves as both a structural support for the tag design and an insulator between the top patch and the ground. The foam substrate has dimensions of *l* × *l* mm² and a thickness of *h*_*s*_ = 3.2 mm. The polyimide layer, illustrated in Fig. 6, has a relative permittivity of 3.5 and loss tangent of 0.0027, which are standard for Kapton at UHF frequencies. It measures 0.05 mm in thickness. Together, these substrates provide structural integrity and slight flexibility to the tag antenna. The conductive layer used is a rolled annealed copper foil with a thickness of 0.009 mm and a conductivity of 5.8 × 10⁷ S/m. These material properties were applied in the full-wave simulation model. Minor differences observed between simulated and measured results are primarily attributed to small tolerances in the material properties particularly the permittivity of the foam.

**Fig. 7:**
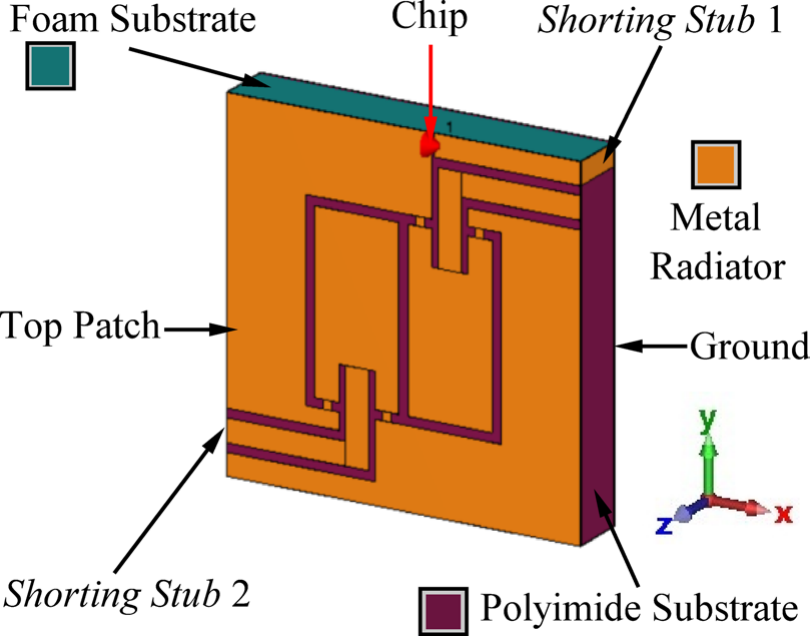
Three-dimensional schematic illustration of the proposed on-metal RFID tag antenna structure.

A UCODE-9 microchip is attached to the top radiating patch, as shown in Figs. 5 and 6. At 915 MHz, the microchip’s input impedance is 13 – *j*191 Ω, and its read sensitivity is − 21.85 dBm. The antenna structure has incorporated two open stubs symmetrically positioned on the top patch, along with a square ground patch. The top and ground patches are shorted through two shorting stubs labelled as *Shorting Stubs* 1 and 2. The polyimide substrate accommodates the top patch, shorting stubs as well as the ground patch, which are folded and attached to the side, bottom, and top surfaces of the foam substrate using thin double-sided adhesive tapes, as depicted in Fig. 5. The adhesive layer has an approximate thickness of 0.05–0.1 mm and a relative permittivity in the range of *ε*_*r*_ ≈ 2–3. Although this layer is electrically thin compared to the foam substrate (*hs* = 3.2 mm), it may introduce slight variations in the effective dielectric environment. The tag is designed to be placed at the center of a 20 cm × 20 cm aluminium plate, with its ground connected to the metal surface. In Figs. 7, and 3D schematic illustration is provided to further clarify the folding structure of the antenna and Table 1 outlines the complete set of geometrical parameters for the proposed antenna design.

Furthermore, the proposed folded-patch structure incorporates two symmetrically positioned open stubs at the upper edges of the radiator. These stubs effectively extend the current path length, thereby lowering the resonant frequency without increasing the overall footprint of the tag. In addition, the open-ended geometry introduces controlled capacitive loading that improves impedance matching between the antenna and the chip, resulting in a broader operational bandwidth. The symmetrical placement of the stubs ensures uniform current distribution across the patch, which stabilizes the radiation characteristics and minimizes polarization distortion. This structural arrangement provides a simple yet efficient means of achieving wideband performance in a compact, single-layer configuration.

**Table 1:** The optimized design parameters of the proposed tag antenna.

Design parameter	Value (mm)	Design parameter	Value (mm)	Design parameter	Value (mm)
*l*	20.0	*s*_2_	5.25	*i*_5_	0.5
*s*	1.2	*i*_1_	16.45	*i*_6_	10.0
*h*	3.32	*i*_2_	1.3	*i*_7_	11.62
*h*_*s*_	3.2	*i*_3_	4.75	*i*_8_	0.5
*s*_1_	6.72	*i*_4_	2.0	–	–

**Fig. 8:**
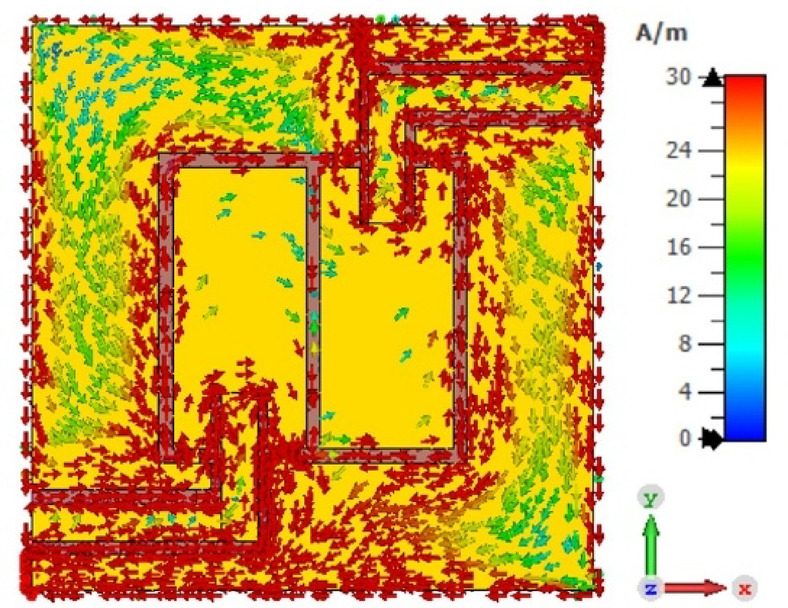
Simulated surface current distributions of the proposed antenna at the resonant frequency.

Next, the operating mechanism of the proposed tag antenna is analyzed through the surface current distribution. Figure 8 presents the simulated surface current distribution of the antenna at the resonant frequency. The proposed electrically small folded square patch antenna operates predominantly in the fundamental TM₀₁ resonance mode^29^, which is identified by the strong current concentration along the radiating edges and folded sections of the patch structure. As shown in Fig. 8, the surface current is mainly distributed along the perimeter of the folded patch and the open stub regions, where the current paths are extended due to the folding configuration. This extended current path effectively increases the electrical length of the antenna without significantly increasing its physical size, enabling the antenna to resonate at the desired UHF RFID operating frequency despite its compact dimensions. The folded geometry and open stubs introduce additional inductive loading, which helps achieve resonance tuning and improves impedance matching with the RFID microchip. It can also be observed that the dominant surface current flows primarily along the *y*-axis direction, indicating a strong alignment of the current distribution along this axis. This directional current flow supports the excitation of the TM₀₁ mode, where the current distribution forms a single half-wave variation along the principal dimension of the patch. The resulting current distribution establishes the fundamental resonance behavior of the antenna and directly influences its radiation characteristics, impedance response, and power transfer efficiency. Furthermore, the strong current concentration near the folded edges and stub regions demonstrates that these structural elements play a critical role in controlling the effective current path and electromagnetic coupling within the antenna structure.

Similar current behavior associated with the TM₀₁ resonance mode has been reported in compact folded patch antennas and on-metal RFID tag designs, confirming the operating mechanism of the proposed antenna configuration^30^. Furthermore, the concentration of current along the *y*-axis also plays a crucial role in determining the polarization of the radiated field, typically resulting in dominant linear polarization. Additionally, this current distribution is very useful for overall radiation efficiency and bandwidth of the design. The combined effect is an increase in both effective inductance and capacitance, which lowers the resonant frequency of the antenna. Similarly, the magnetic field distribution forms closed-loop patterns around the surface current paths, indicating the presence of strong magnetic coupling along the folded current path. The magnetic field is primarily concentrated around the conducting strips and stub regions, confirming the circulating current behavior associated with the TM₀₁ mode. Together, the observed surface current flow, electric field concentration, and magnetic field loops confirm that the proposed antenna operates predominantly in the fundamental TM₀₁ resonance mode, which governs the radiation characteristics, impedance behavior, and overall electromagnetic performance of the antenna.

**Fig. 9:**
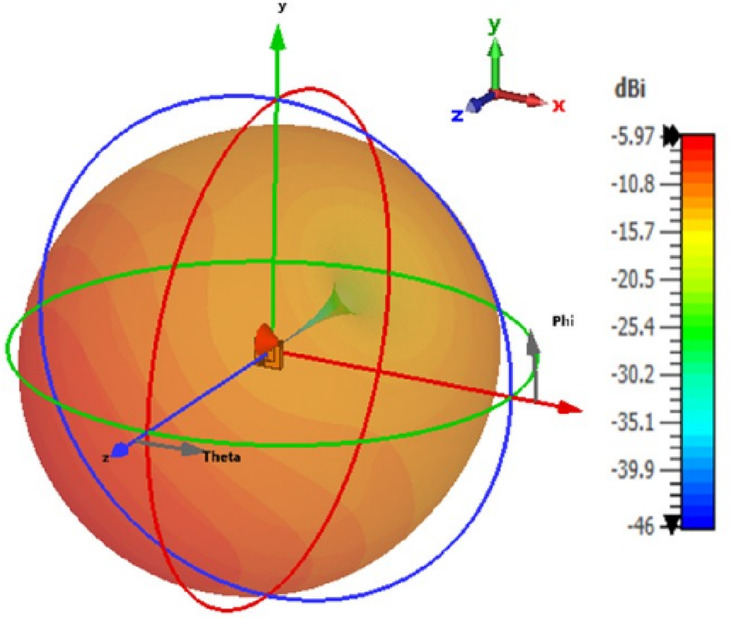
Simulated three-dimensional radiation pattern of the proposed tag antenna at the resonant frequency of 916 MHz.

This principle is well established in antenna theory, where electrically longer current paths and stronger capacitive interactions shift the resonant frequency downward^31^. Additionally, the strong surface current distribution along the open stubs introduces inductive and capacitive loading. The asymmetric current flow and strategically positioned middle square slot structure contribute to parasitic effects, generating phase shifts that enhance both directional radiation and bandwidth, making this design highly suitable for UHF RFID applications. Figure 9 shows the simulated 3D radiation pattern of the proposed tag antenna at its resonant frequency of 916 MHz, as obtained from CST Studio Suite. The antenna exhibits a directional radiation pattern, which is typical for folded patch designs placed on metal surfaces. At this frequency, the antenna achieves a maximum realized gain of − 5.97 dBi, corresponding to a theoretical calculated read range of approximately 9 to 10 m under ideal conditions with a regulated reader power of 4 W EIRP. The theoretical maximum read range, *R*, was computed using the Friis transmission equation Eq. (1):

1 $$\:R=\left(\frac{\lambda\:}{4\pi\:}\right)\sqrt{\frac{{P}_{EIRP}\cdot\:{G}_{tag}}{{P}_{chip}}}$$

where *P*_*EIRP*_ corresponds to the effective isotropic radiated power (4 W EIRP). The realized gain, *G*_*tag*_, was determined using the expression *G*_*tag*_ = *P*_*chip*_ / (*L*_*T*_ × *P*_*tx*_), where *L*_*T*_ accounts for cumulative cable and free-space losses, *P*_*chip*_ denotes the chip’s read sensitivity (–21.85 dBm), and *P*_*tx*_ represents the transmitted power at a given frequency. The theoretical read range is estimated using the RFID Friis transmission model, which incorporates the chip sensitivity, antenna realized gain, and power transmission coefficient. Unlike conventional antenna link budget calculations, the read range in passive RFID systems is strongly influenced by the chip activation threshold. The realized gain used in this formulation inherently includes radiation efficiency and mismatch losses.

**Fig. 10:**
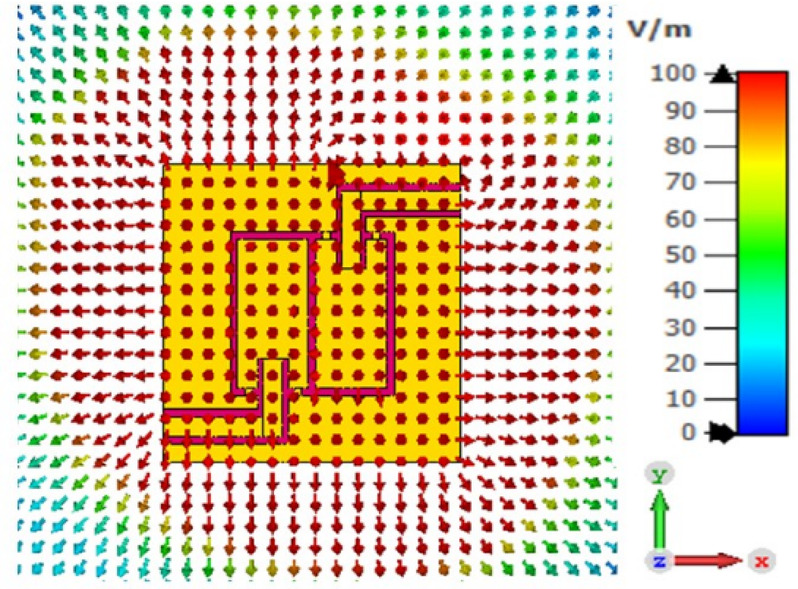
Simulated electric field distributions of the proposed tag antenna at the resonant frequency.

**Fig. 11:**
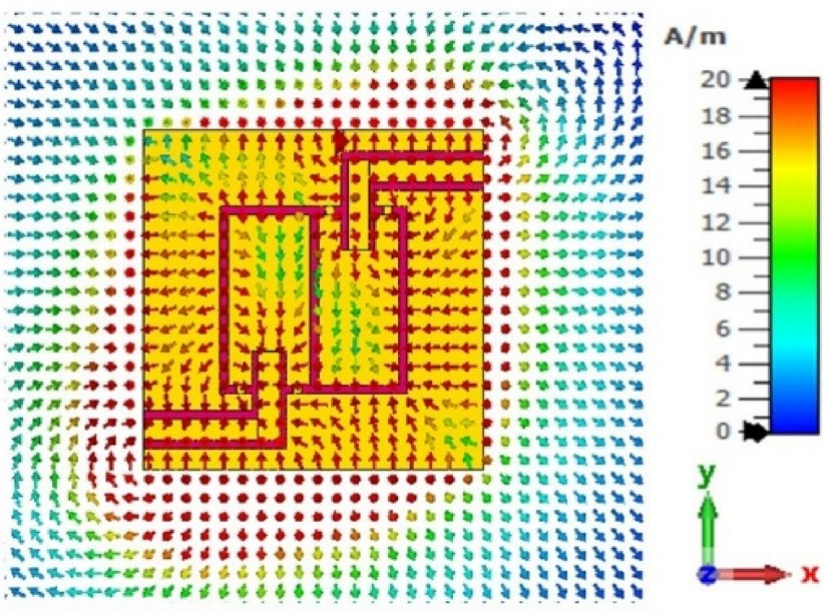
Simulated magnetic field distributions of the proposed tag antenna at the resonant frequency.

Figures 10 and 11 illustrate the electric and magnetic fields, offering enhanced clarity and deeper insight into the antenna’s behavior. Notably, the electric field distribution remains uniform and stable along the patch surface, supporting consistent broadside radiation, as confirmed by the simulated field results in Figure. 10. Meanwhile, the magnetic field is strongest along the edges, generating a strong magnetic dipole moment, as illustrated in Fig. 11. This field consistency enhances performance and ensures a stable broadside radiation pattern, perpendicular to the patch surface. Despite this, the antenna maintains stable and evenly distributed surface current oscillations across the patch. These oscillations, which are periodically aligned with the folded geometry, ensure a consistent current flow and a stable interaction between inductive and capacitive effects. This stability enhances impedance matching and supports uniform field distribution, as illustrated in the simulated electric and magnetic field plots (Figs. 10 and 11). As a result, the antenna achieves improved resonance behavior and an enhanced operational bandwidth across the UHF band (855–942 MHz).

### Parameter analysis and equivalent circuit model

To better understand the tunability and impedance behavior of the proposed tag antenna, a detailed parametric analysis was performed by varying key structural parameters. Specifically, *i*_2_ (open stub width) and *s* (shorting stub width) were selected due to their direct influence on the antenna’s reactive loading and impedance matching. These elements enable practical fine-tuning of the resonant frequency without altering the overall tag dimensions. The open stubs introduce both inductive and capacitive loading, which help fine-tune the resonant frequency and improve impedance matching with the RFID chip. At the same time, the shorting stubs govern the current return path between the top patch and the ground, making them equally critical to the antenna’s overall performance. These two elements act together to shape the resonance characteristics and directly influence the power transfer coefficient (*τ*). The effect of the shorting stubs can be better understood in terms of their width: a wider stub reduces inductive impedance, providing a more efficient current return path. This shortens the effective current length, which in turn shifts the resonant frequency upward. Conversely, a narrower stub increases inductive impedance, forcing the current to travel a longer effective path, thereby lowering the resonant frequency. This tunability enables precise control of the antenna’s electrical characteristics without altering its overall dimensions, while maintaining a consistently high power transfer efficiency. Additionally, an equivalent circuit model was developed to provide a simplified electrical representation of the antenna. Each circuit element corresponds to physical components of the design, allowing for rapid evaluation and insight into the antenna’s performance. This combined analytical approach facilitates effective optimization and supports the accuracy of the full-wave simulation results.

**Fig. 12:**
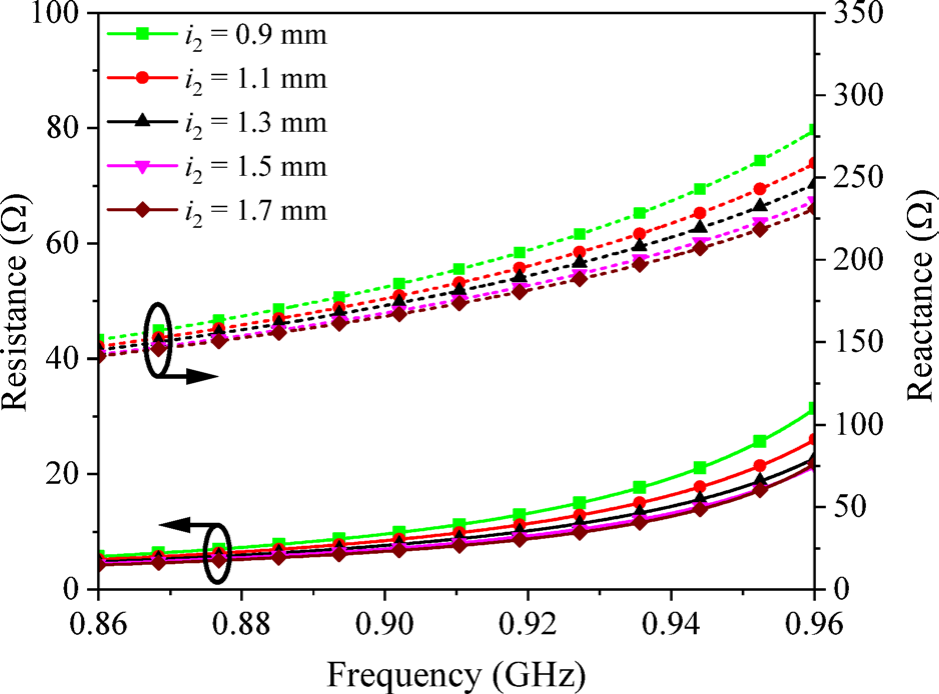
Effect of varying the open stub width (*i*_*2*_) on the input impedance of the proposed RFID tag antenna.

**Fig. 13:**
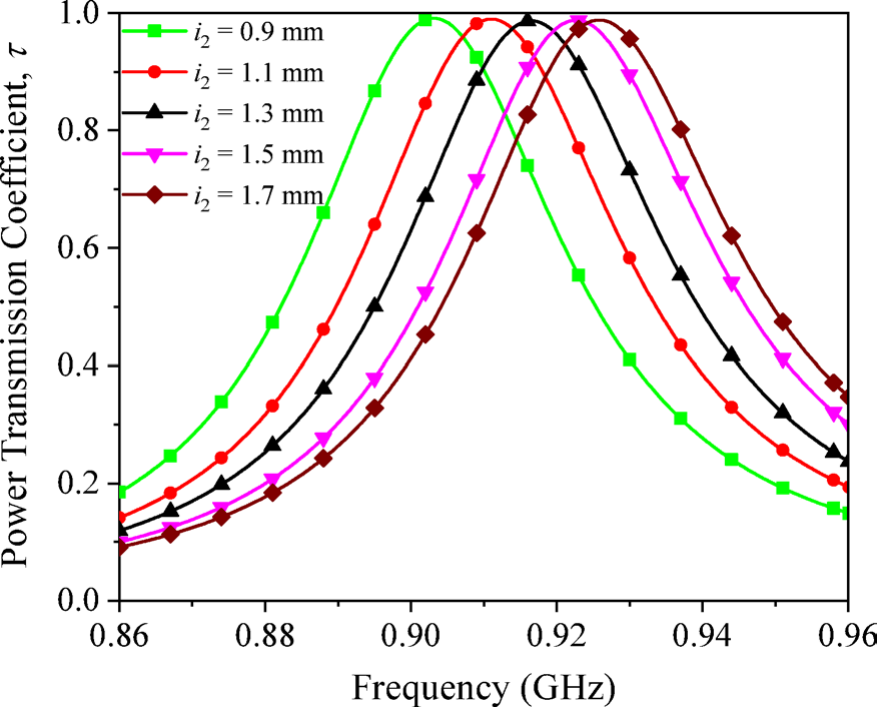
Effect of varying the open stub width (*i*_2_) on the simulated power transmission coefficient (PTC, *τ*) of the proposed tag antenna.

**Fig. 14:**
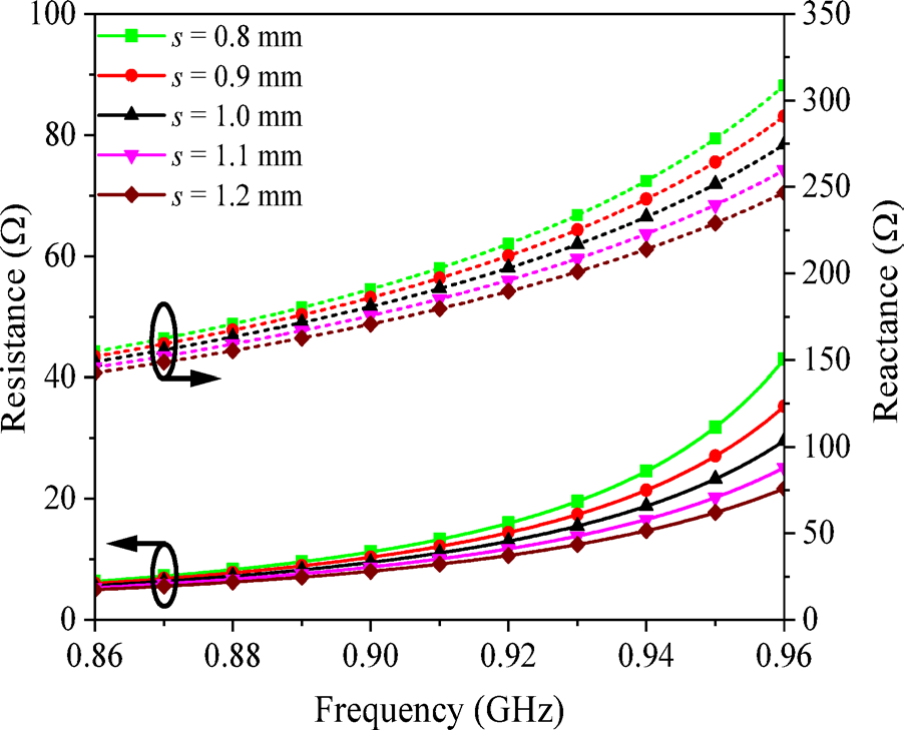
Effect of varying the shorting stub width (*s*) on the simulated input impedance of the proposed tag antenna.

**Fig. 15:**
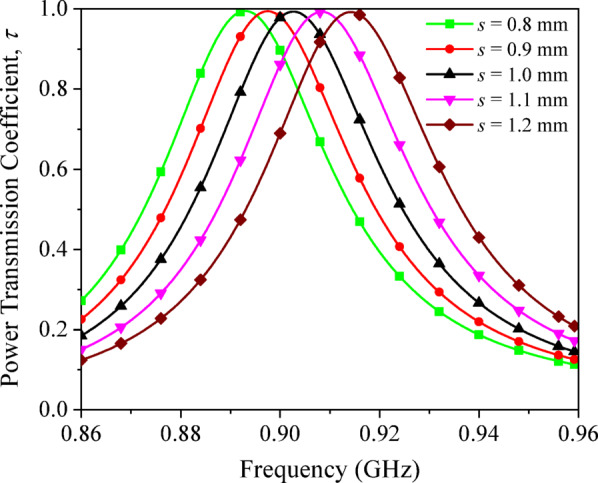
Effect of varying the shorting stub width (*s*) on the simulated power transmission coefficient (PTC, *τ*) of the proposed tag antenna.

Figures 12 and 13 illustrate the impact of varying *i*_2_ on the resonant frequency and performance of the tag antenna. As *i*_2_ increases from 0.9 mm to 1.7 mm in 0.2 mm increments, the resonant frequency shifts upward from 900 MHz to 930 MHz, at a rate of approximately 6 MHz per step. This indicates that reducing *i*_2_ enhances the antenna’s inductance. Additionally, Fig. 13 demonstrates that the power transmission coefficient (PTC) remains consistently high, at around 98%, across all configurations. The parameter *i*_2_ proves effective for fine-tuning the resonant frequency of the tag without degrading *τ*. The dual open stubs introduce sufficient resistance and reactance into the antenna, enabling conjugate matching with the chip. This straightforward tuning mechanism eliminates the need for external components such as lumped elements or vias. Figures 14 and 15 illustrate the impact of altering the width (*s*) of the shorting stubs on the performance of the proposed tag antenna. When *s* is increased from 0.8 mm to 1.2 mm, the resonant frequency of the proposed tag antenna shifts upward by approximately 6 MHz. Figure 15 shows that the power transmission coefficient (PTC) remains consistently around 98% across all variations. Modifying the width of the shorting stubs offers an effective approach for fine-tuning the tag’s resonant frequency. In addition, variations in the adhesive layer thickness and bonding uniformity may lead to small shifts in the resonant frequency due to changes in the effective dielectric spacing between the radiating patch and ground. However, the proposed antenna demonstrates tolerance to such variations, as indicated by the parametric analysis, where stable impedance matching and high-power transmission coefficient are maintained over a range of structural parameters. Therefore, the antenna performance is expected to remain stable under typical manufacturing tolerances.

**Fig. 16:**
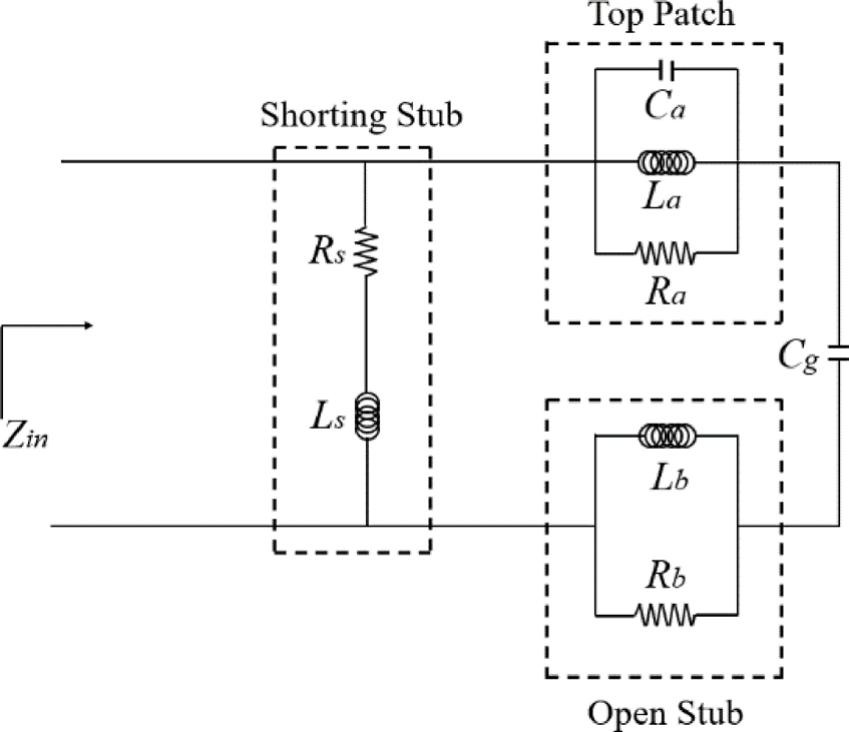
Equivalent circuit model (*R*_*s*_ = 2.31 Ω, *L*_*s*_ = 4.25 nH, *R*_*a*_ = 9.32 kΩ, *L*_*a*_ = 5.87 nH, *C*_*a*_ = 5.62 pF, *C*_*g*_ = 5.41 pF, *R*_*b*_ = 5.1 Ω, *L*_*b*_ = 13.2 nH).

The electrical properties of the tag antenna can be analyzed using the equivalent circuit model shown in Fig. 16. This model offers a simplified electrical representation of the patch antenna, providing a clearer understanding of the interactions between different resonator components and the impact of parasitic effects. By utilizing the equivalent circuit, designers can gain quick physical insights into antenna behavior, aiding in performance optimization. Furthermore, employing circuit simulations significantly accelerates the design process, as they generate results much faster than full-wave CST simulations, allowing for efficient parameter tuning and validation. The proposed equivalent circuit model is primarily derived based on analytical formulations, as presented in this work. However, for certain parameters such as the patch resistances $\:{R}_{a}\:$and $\:{R}_{b}$, a macro-model representation is adopted, as it is difficult to obtain accurate closed-form expressions for electrically small patch antennas. These parameters are therefore determined to represent the overall loss and coupling effects while maintaining a physically meaningful model.

The circuit parameters are obtained using a hybrid approach, where analytically derived values are combined with parameter extraction to achieve good agreement between the circuit model and full-wave simulation results, particularly in terms of input impedance and resonance characteristics. This hybrid analytical extraction method is widely used for electrically small antenna modeling. The equivalent circuit model is valid over the operating frequency range around the resonance, specifically within the UHF RFID band (902–928 MHz), where the antenna exhibits its intended performance. Outside this range, the accuracy of the model may reduce due to higher-order and distributed effects not captured by the simplified circuit representation. In this configuration, shorting stubs are used to connect the top patch to the ground. Resistance (*R*_*a*_), inductance (*L*_*a*_), and capacitance (*C*_*a*_) are the lumped components that represent the top radiating patch (*Z*_*a*_). The inductance (*L*_*a*_) of the top patch can be calculated using the Eq. (2)^32^.

2 $$\:{L}_{a}=200\left({i}_{7}+I\right)\left\{\mathrm{ln}\left[\frac{2\left({i}_{7}+I\right)}{0.5\left(l+l\right)+{C}_{t}}\right]+0.50049+\frac{\left(0.5\left(l+l\right)+{C}_{t}\right)}{3\left({i}_{7}+I\right)}\right\}\:\mathrm{n}\mathrm{H}$$

In Eq. (2), $\:({i}_{7}+I)$denotes the effective conductor length of the folded patch, where $\:{i}_{7}\:$is the vertical segment of the top patch and $\:I=l-{i}_{7}=8.38\mathrm{\:mm}\text{}$represents the extension length. The denominator term $\:0.5\left(l+l\right)\:$is defined as the effective conductor width and length, obtained by averaging the contributions from both sides of the patch. The formula for capacitance *C*_*a*_ = *ε*_*o*_*ε*_*r*_*A*_*e*_/*h*_*s*_^33^ can be utilized for calculating capacitance; in this formula, *ε*_*r*_ and *h*_*s*_ represent the relative permittivity and thickness of the foam substrate, respectively. *A*_*e*_ = (*i*_7_ + *I*)(*l* + *l*)/2 is the patch’s effective area. In this expression, $\:\left({i}_{7}+I\right)\:$represents the effective conductor length of the folded patch, while $\:\left(l+l\right)\:$accounts for the total width and length contribution from both sides of the conductor. The factor of $\:1/2\:$is applied to average the overlapping contributions and avoid double-counting of the conductor width. This approximation provides a practical estimate of the effective patch area. The resistance (*R*_*a*_) can be determined using a macro model method, as described in reference^34^. The lumped components resistance (*R*_*b*_) and inductance (*L*_*b*_) can be utilized for describing the two open stubs (*Z*_*b*_). The open stubs can be represented as inductors, with a stub length of *L*_1_ = *s*_1_ + *s*_2_ = 11.97 mm, stub width of *i*_2_ = 1.3 mm, and the thickness of the copper layer is *C*_*t*_ = 0.009 mm. The inductance (*L*_*b*_) for each stub is calculated using the Eq. (3)^35^.

3 $$\:{L}_{b}=2{L}_{1}\left\{\mathrm{l}\mathrm{n}\left[\frac{2{L}_{1}}{{i}_{2}+{C}_{t}}\right]+0.50049+\frac{\left({i}_{2}+{C}_{t}\right)}{3{L}_{1}}\right\}\mathrm{n}\mathrm{H}$$

The resistance (*R*_*b*_) can be found using a macro model method. Here, a serial connection between a resistance (*R*_*s*_) and an inductance (*L*_*s*_) represents a shorting stubs. *R*_*s*_ = 2[(*ρh*)/(*s* × *h*_*s*_)] *F*(*x*) *K*_*c*_^36^ is the resistance of the shorting stubs. Here, *x* = [2(1 + *h*_*s*_/*s*)] (*δ* /*h*_*s*_), where *δ* = 2.18 × 10^− 6^ m is the copper skin depth, and the resistivity of the copper is *ρ* = 1.72 × 10^− 8^ Ωm. The current crowding factor is *K*_*c*_ = 1.94. The inductance (*L*_*s*_) of the shorting stubs can be determined using the following Eq. (4).

4 $$\:{L}_{s}=400h\left\{\mathrm{l}\mathrm{n}\left[2h\left({C}_{t}+s\right)\right]+0.50049+\frac{\left({C}_{t}+s\right)}{3h}\right\}\mathrm{n}\mathrm{H}$$

The top patch slot capacitance (*C*_*g*_) can be calculated using the following Eq. (5).

5 $$\:{C}_{g}=\frac{{\epsilon\:}_{0}{\epsilon\:}_{r}{L}_{g}}{2\pi\:}\left\{\mathrm{l}\mathrm{n}\left[0.25+{\left(\frac{{h}_{s}}{{i}_{8}}\right)}^{2}\right]+\left(\frac{{i}_{8}}{{h}_{s}}\right){\mathrm{t}\mathrm{a}\mathrm{n}}^{-1}\left(\frac{2{h}_{s}}{{i}_{8}}\right)\right\}\:\mathrm{p}\mathrm{F}$$

^37^, (Eq. 2.6), where the total slot length *L*_*g*_ = 3*i*_6_ + 2*i*_3_ + 2*s*_1_ + 2(*s*_1_ + *i*_2_ + *i*_8_) + 2(*s*_2_ + *i*_8_ ) + 2(*s*_2_ – *i*_2_). The values of all calculated elements are provided in the figure caption, facilitating an understanding of the tag antenna’s characteristics. The total input impedance (*Z*_*in*_) of the proposed tag antenna can then be described using Eq. (6). Equations (7)–(9) represent the lumped-element modeling of the individual components of the proposed equivalent circuit. Specifically, Eq. (7) corresponds to the top patch, which is represented as a parallel RLC circuit to account for its resonant behavior and associated losses. Equation (8) models the open stubs as a parallel combination of resistance and inductance, capturing both the radiation resistance and the inductive loading effect introduced by the stub geometry. Furthermore, Eq. (9) represents the shorting stubs using a series RL network, where the resistance models the conduction and ohmic losses while the inductance accounts for the current path introduced by the shorting stubs.

It should be noted that the proposed equivalent circuit model provides a simplified representation of the antenna behavior near the operating resonance. The lumped parameters were obtained using a combination of geometry-based analytical expressions and impedance-based interpretation of the antenna structure and were validated through comparison with the simulated and measured input impedance. Although the open stubs introduce both inductive and capacitive effects in the folded structure, they are modelled using a parallel RL branch to capture their dominant impedance contribution near resonance while maintaining a compact and physically interpretable equivalent circuit model. By formulating each structural element in this manner, the overall equivalent input impedance *Z*_*in*_ of the tag antenna can be systematically derived through appropriate series and parallel combinations.

**Fig. 17:**
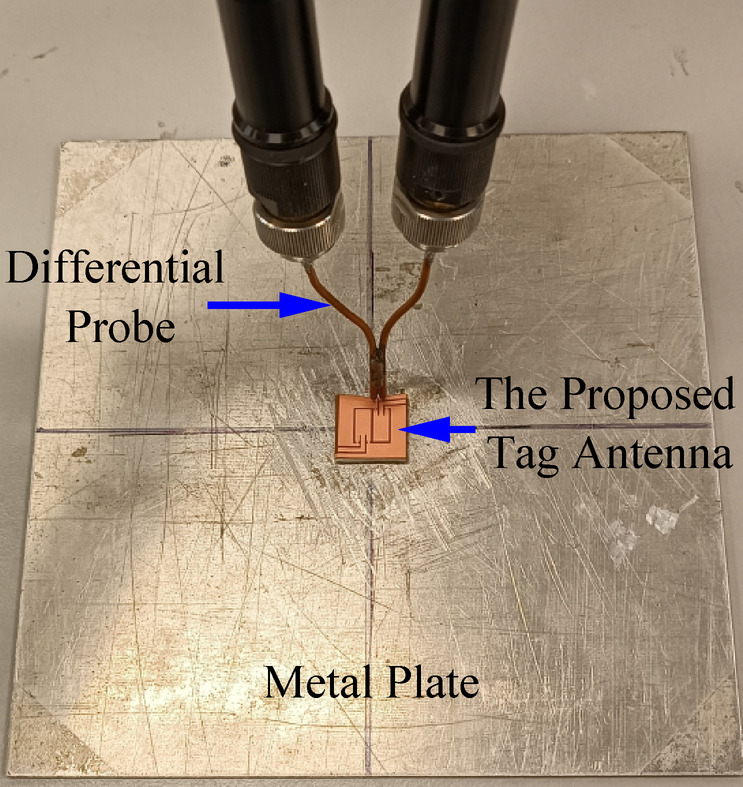
Impedance measurement setup of the proposed RFID tag antenna using a vector network analyzer (VNA).

**Fig. 18:**
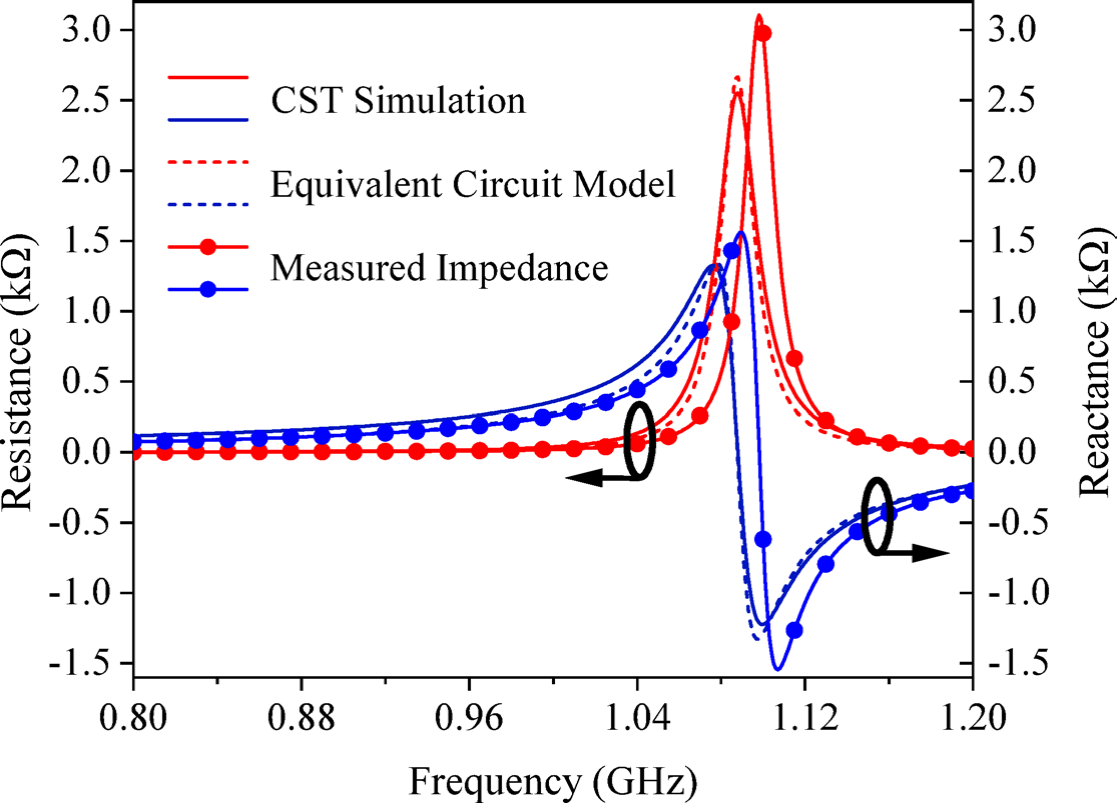
Comparison between the modeled and simulated input impedances of the proposed RFID tag antenna.

The equivalent circuit model assists in determining the antenna’s impedance, a crucial aspect in tag design to achieve a conjugate matching condition (*Z*_*in*_ = Z*_chip_), ensuring optimal power transfer. After removing the UCODE-9 microchip, a specially assembled differential probe is used to access the solder pads across the antenna port. A Rohde & Schwarz ZNB Series Vector Network Analyzer (VNA) was used to measure the input impedance of the tag antenna after the removal of the chip. A custom-designed differential probe was used for this measurement and was calibrated using the Short-Open-Load (SOL) method. Calibration standards, fabricated on a substrate with matching material properties and geometry, were employed to accurately establish the reference plane at the probe tips. This procedure enabled effective de-embedding of probe-related parasitics, ensuring precise impedance measurements. The measurement setup, including the differential probe connected to the VNA, is illustrated in Fig. 17. Figure 18 compares the antenna impedances obtained from the CST full-wave simulation (solid curves) with those from the equivalent circuit model (dashed curves) and measured impedance (dash dot dash curves). The slight differences observed in the curves can be attributed to assumptions made during the calculation of component values, such as the assumption of a uniform current distribution across the radiating patches. However, the equivalent circuit is still helpful for analyzing impedance. To further evaluate the accuracy of the equivalent circuit model, a quantitative comparison with the CST full-wave simulated impedance was performed. The results show that the deviation between the modelled and simulated impedance remains within approximately 5–10% near the resonance frequency, indicating good agreement in the operating region. Away from resonance, the deviation increases to approximately 10–20%, which can be attributed to distributed electromagnetic effects, fringing fields, and the simplified lumped-element approximation. Therefore, the equivalent circuit model is most accurate in the vicinity of the resonance region and is primarily intended for physical interpretation and design optimization.

6 $$\:{Z}_{in}=\left({Z}_{a}\left(2{Z}_{b}\right)+\frac{1}{j{C}_{g}}\right)\:{\left|\right|\:Z}_{s}$$

where

7 $$\:{Z}_{a}=\frac{j\omega\:{R}_{a}{L}_{a}}{{R}_{a}-{\omega\:}^{2}{R}_{a}{L}_{a}{C}_{a}+j\omega\:{L}_{a}}$$

8 $$\:{Z}_{b}=\frac{j\omega\:{R}_{b}{L}_{b}}{{R}_{b}-{\omega\:}^{2}{R}_{b}{L}_{b}+j\omega\:{L}_{b}}$$

9 $$\:{Z}_{s}={R}_{s}+j\omega\:{L}_{s}$$

### Tag fabrication and measurement setup

**Fig. 19:**
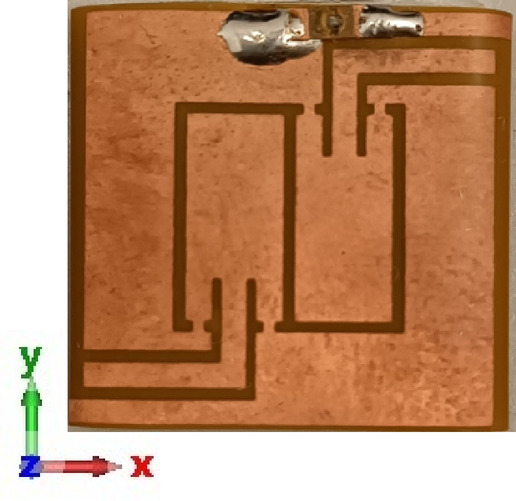
Photograph of the fabricated prototype of the proposed RFID tag antenna.

**Fig. 20:**
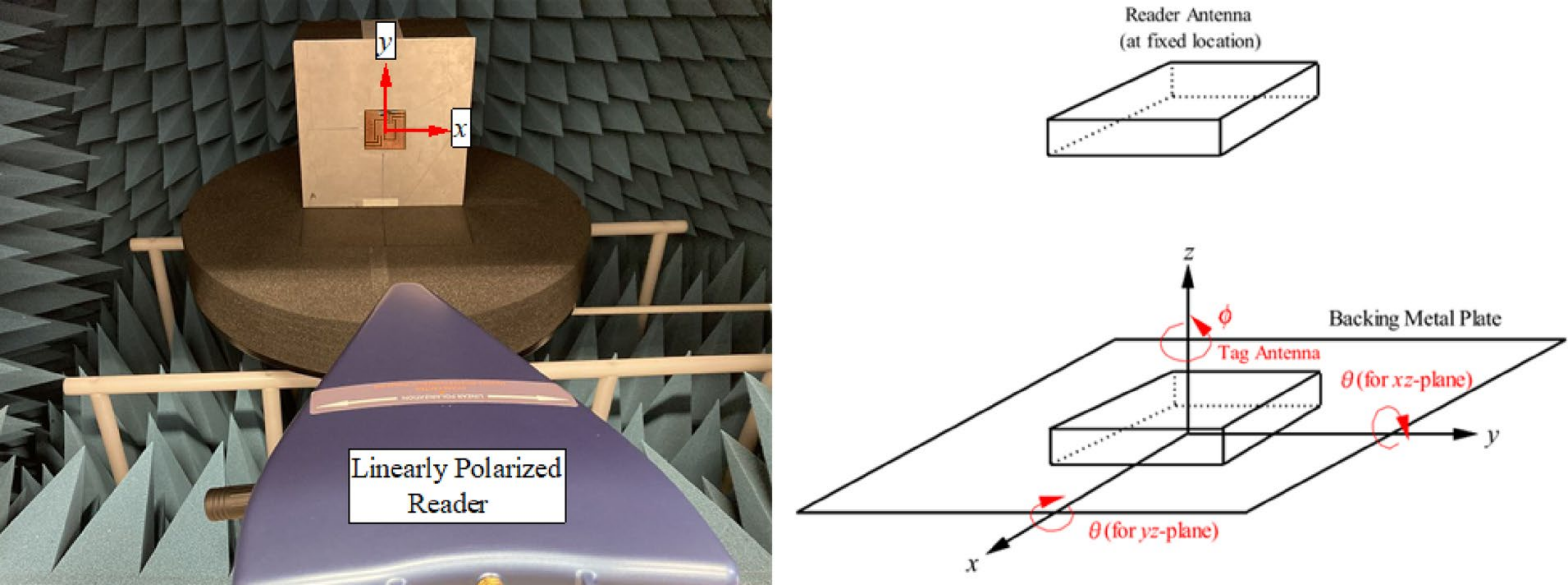
Measurement setup inside the anechoic chamber for read-range evaluation and definition of the planes used for measuring the read patterns.

The performance of the fabricated tag antenna, designed using the optimized parameters listed in Table 1, was evaluated using a Voyantic Tagformance Pro RFID measurement system^38^. The tag prototype, with the UCODE-9 microchip soldered across a narrow slit (Fig. 19), was mounted at the center of a 20 cm × 20 cm aluminum plate to emulate practical on-metal deployment conditions. All measurements were conducted in a shielded anechoic chamber to minimize external interference. The measurement setup employed a linearly polarized UHF RFID reader antenna, positioned at a fixed distance of 52 cm from the tag under test, measured from the antenna aperture to the center of the tag, and operated in accordance with the manufacturer’s guidelines for far-field RFID measurements. The measurements were conducted using a UHF wideband patch antenna (standard Tagformance antenna) operating over the 600–1300 MHz range, with a typical gain of 8.5 dBi. The measurements were conducted using a linearly polarized reader antenna, and the tag was aligned with the same polarization direction to ensure maximum power transfer; any polarization mismatch may result in reduced read range. The transmitted power is calibrated in the Tagformance system by considering the reader antenna gain and cable losses, ensuring that the effective isotropic radiated power (EIRP) is maintained at 4 W, in accordance with standard UHF RFID measurement conditions. During the measurements, the tag was centrally mounted on the metallic plate and supported using a Styrofoam fixture to maintain consistent alignment and spacing. Prior to testing, a commercially calibrated reference tag was used to determine the forward-link path loss and calibrate the system. The Tagformance Pro system was then used to measure the tag’s read sensitivity, realized gain, and read range. To evaluate the radiation characteristics, the tag assembly was rotated incrementally, allowing the read pattern to be recorded across multiple planes, as illustrated in Fig. 20, while the surrounding environment was kept free of nearby scattering objects to minimize measurement interference.

The realized gain (*G*_*r*_) is a critical parameter in evaluating the performance of RFID tag antennas, and it is calculated using the equation Eq. (10)^34^:

10 $$\:{G}_{r}=\frac{{P}_{c}}{{L}_{l}\times\:{P}_{t}}$$

**Fig. 21:**
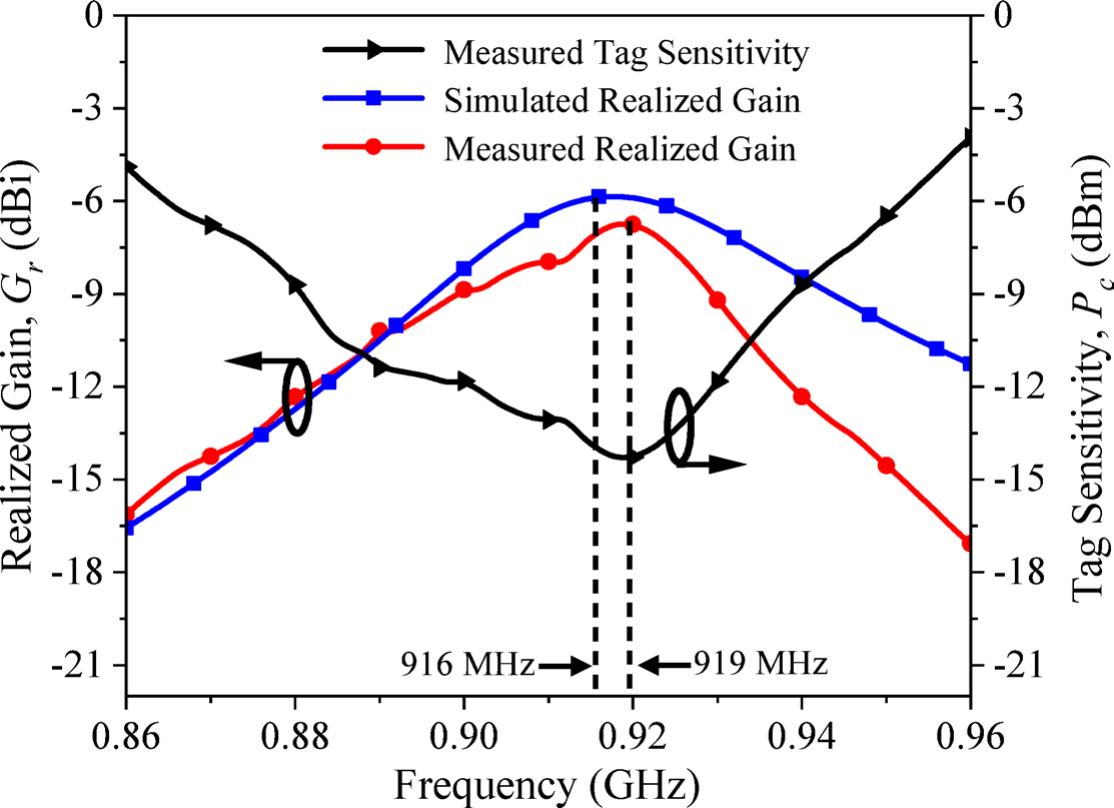
Measured tag sensitivity together with the simulated and measured realized gains of the proposed RFID tag antenna.

Here,* P*_*c*_ represents the read sensitivity of the UCODE-9 microchip, which is specified as − 21.85 dBm. The term * P*_*t*_ denotes the threshold power of the RFID reader at a particular frequency during the threshold power sweep, while * L*_*l*_ accounts for the forward-link loss, encompassing both the free-space path loss and any additional losses introduced by cables or connectors in the system. Prior to performing measurements, a reference tag is employed to calibrate the RFID system. This step ensures an accurate determination of the path loss by accounting for all components in the measurement setup, including environmental factors and system imperfections. This calibration process is essential for obtaining reliable and reproducible data. The comparison between the simulated and measured realized gains is presented in Fig. 21. At the frequency of 919 MHz, the maximum measured realized gain (*G*_*r*_) is observed to be − 6.71 dBi and the measured tag sensitivity is − 14.31 dBm. The results shows a close agreement between the simulated and measured data, highlighting the accuracy of the design and modeling approach. The consistency between these results validates the simulation methods and confirms that the fabricated tag performs as intended within the operating frequency range. This alignment also underscores the reliability of the measurement setup and the accuracy of the calibration procedure used in the analysis.

**Fig. 22:**
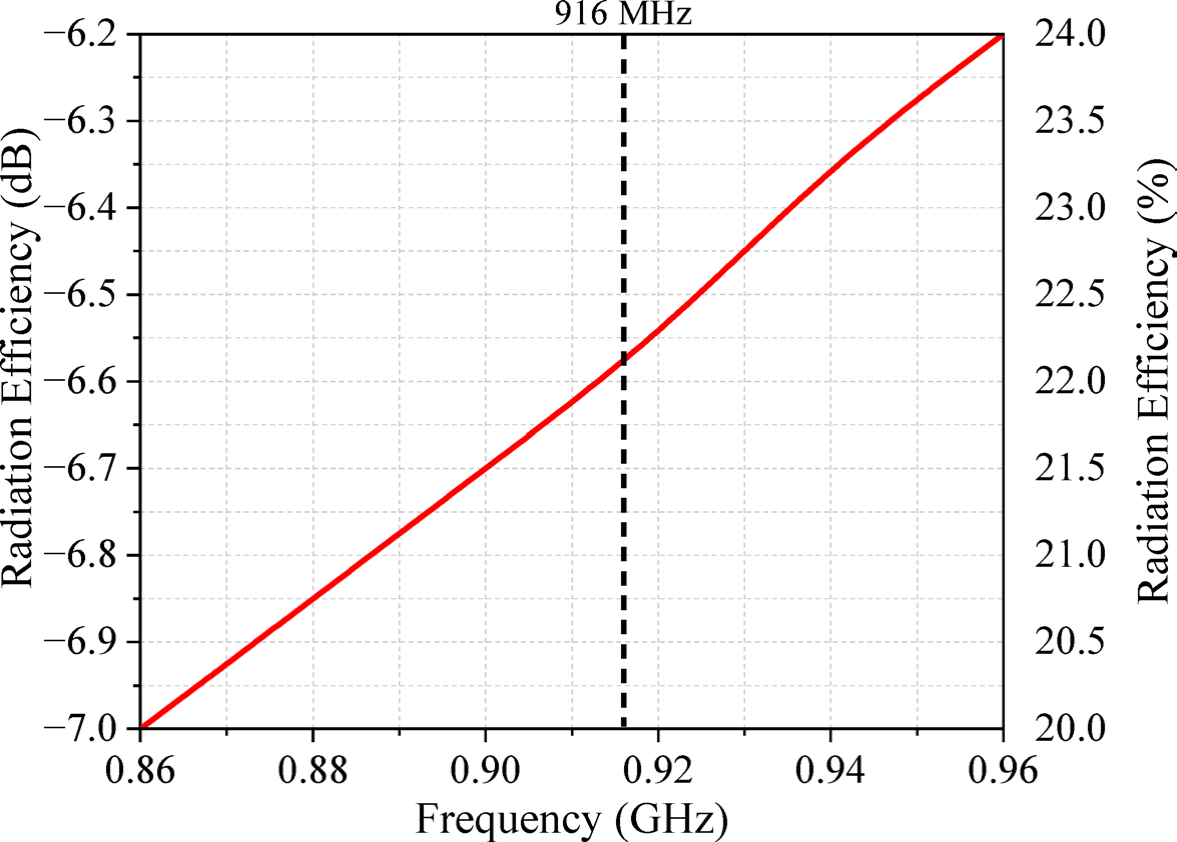
Simulated radiation efficiency of the proposed RFID tag antenna expressed in both dB and percentage (%) scales.

As shown in Fig. 22, the simulated radiation efficiency of the proposed tag antenna increases gradually across the UHF RFID band. The antenna is excited using a discrete port with conjugate matching to the chip impedance, and the reported radiation efficiency is defined in CST as the ratio of radiated power to accepted input power at the port. The radiation efficiency ranges from − 7.0 dB at 860 MHz to − 6.2 dB at 960 MHz, indicating that the antenna maintains a reasonably consistent performance over the entire operating bandwidth. At the center frequency of 916 MHz, the antenna achieves a radiation efficiency of approximately − 6.57 dB. The upward trend confirms that the antenna is capable of sustaining acceptable radiation efficiency levels throughout the wideband operation, making it suitable for practical RFID applications. This level of radiation efficiency is consistent with other compact metal-mountable tag designs, where miniaturization and ground-plane loading typically result in reduced radiation efficiency compared to larger planar structures. Although the simulated radiation efficiency shown in Fig. 22 is moderate (approximately − 6.6 dB, corresponding to about 22% radiation efficiency at 916 MHz), such performance is typical for compact metal-mounted RFID tag antennas where antenna miniaturization and strong coupling with the metallic ground plane introduce additional losses.

**Table:** 

Frequency (MHz)	Directivity (dBi)	Radiation efficiency (%)	Realized gain (dBi)
916	2.9	22	−5.97

## Results

### Read range and orientation measurements

**Fig. 23:**
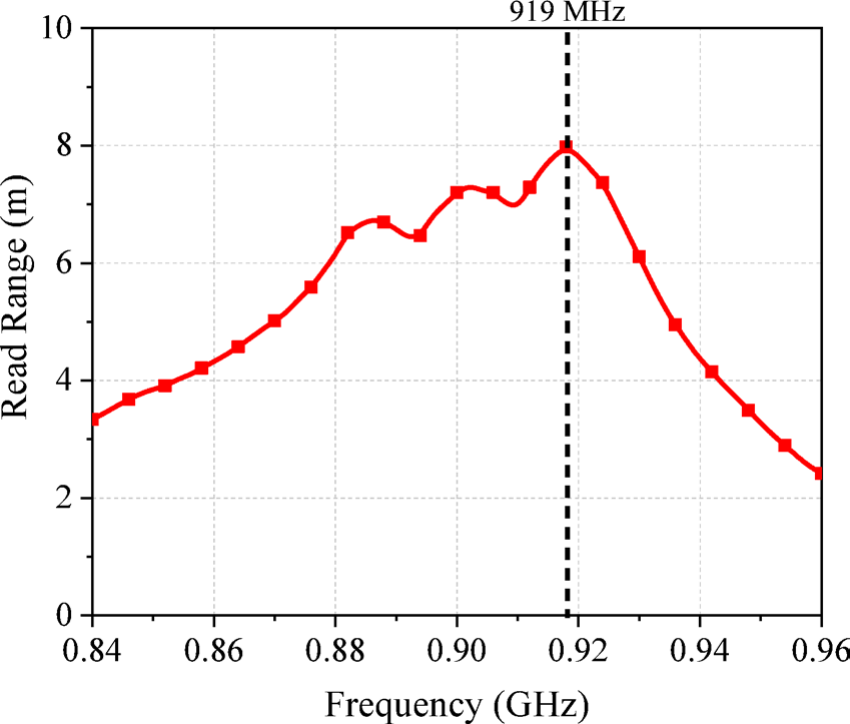
Measured read-range performance of the fabricated RFID tag antenna prototype.

As shown in Fig. 23, the proposed tag has a maximum read range of 7.9 m in the elevation plane (*θ* = 0°) at 919 MHz, when being used on a 20 cm × 20 cm aluminium metal plate. Since the proposed tag antenna is specifically designed for application on metallic surfaces. There are some minor deviations in frequency and read range, as compared to the simulated values, which are caused by the fabrication tolerances resulting from the use of double-sided tape to assemble the tag structure. It should be noted that the reported read range is obtained through calibrated measurements using the Voyantic Tagformance Pro system, where forward-link losses are compensated using a reference tag. Therefore, the measured read range reflects the actual activation distance of the RFID chip under a regulated EIRP of 4 W, rather than a purely analytical estimation based on simplified Friis calculations. Additionally, the antenna was optimized using the nominal bare-die UCODE-9 impedance of 13 − *j*191 Ω at 915 MHz reported in the manufacturer datasheet. However, in the fabricated prototype, a commercial interposer-based packaged version of the chip was used, which was soldered across a narrow slit, as shown in Fig. 19. Such packaging introduces additional parasitic elements, mainly an added capacitance associated with the strap/interposer geometry and a smaller series inductance due to the connection path. Although the packaged-chip impedance was not measured separately in this work, these parasitics are expected to be of first-order importance for compact UHF RFID tags and can perturb the conjugate matching condition, thereby slightly reduce the peak power transmission coefficient (PTC) and shift the measured resonance/read-range peak relative to simulation. This effect contributes to the small discrepancy between simulated and measured results.

**Fig. 24:**
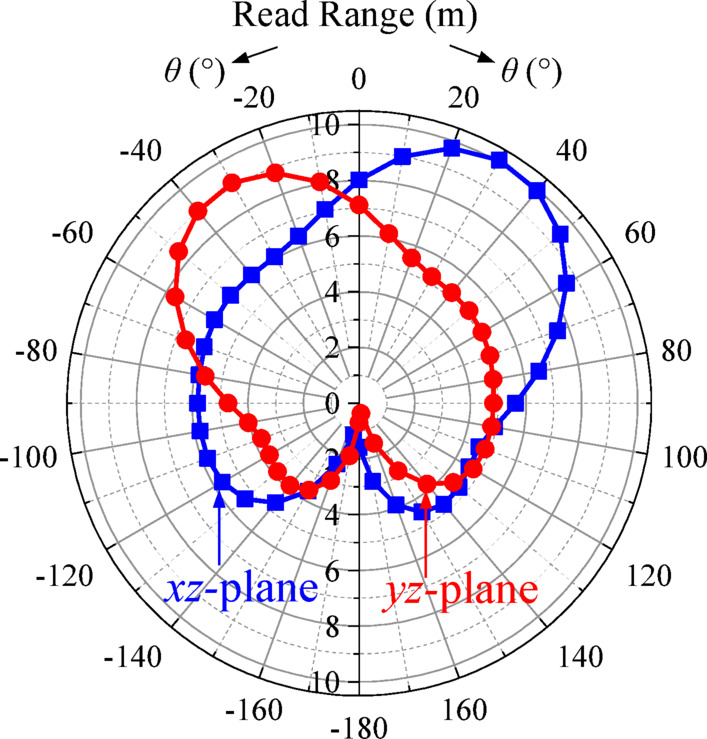
Measured read-range radiation patterns of the fabricated tag antenna in the *xz*- and *yz*-planes.

**Fig. 25:**
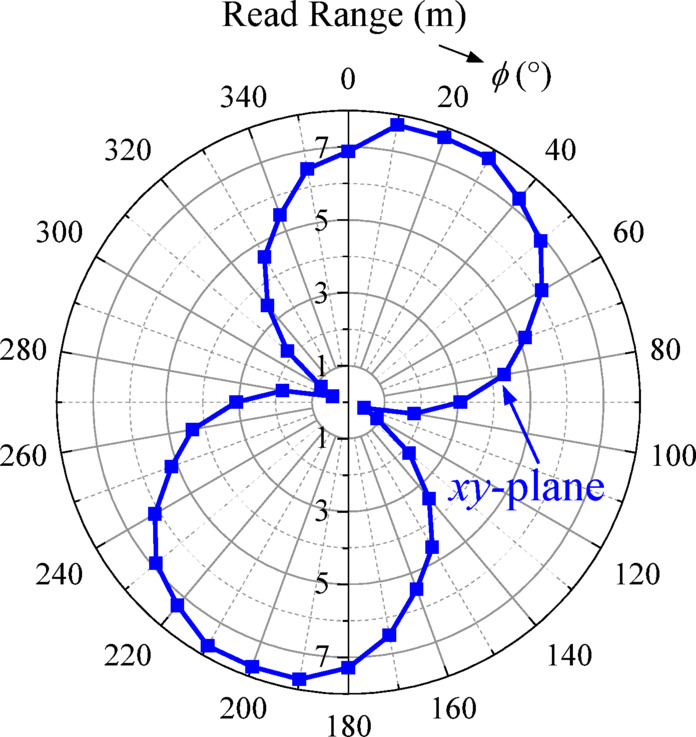
Measured read-range radiation pattern of the fabricated tag antenna in the *xy*-plane.

To gain deeper insight into the real-world performance of the proposed tag antenna, its angular read characteristics were thoroughly evaluated. Measurements were conducted in the *xz* and *yz* planes at the antenna’s resonant frequency of 919 MHz, corresponding to the elevation planes relative to the metal mounting surface. As illustrated in Fig. 24, the maximum measured read range at boresight (*θ* = 0°) is approximately 7.9 m at 919 MHz, while higher values approaching 10 m are observed at off-boresight angles (*θ* ≈ ±30°) due to the antenna’s directional radiation characteristics. In addition, small differences between calculated, simulated, and measured values are caused by practical factors such as fabrication tolerances, the use of a packaged interposer-based chip instead of the bare-die impedance model used in simulation, and measurement uncertainties. These effects slightly alter the impedance matching and radiation performance, leading to variation in the measured read range. As shown in Fig. 25, the measured radiation pattern in the *xy*-plane demonstrates that the proposed tag antenna achieves a maximum read range of approximately 8 m at $\:\phi\:={30}^{\circ\:}$and $\:\phi\:={210}^{\circ\:}$. The symmetric peaks in opposite directions confirm that the antenna exhibits linear polarization. Since a linearly polarized reader antenna was used during the measurements, the measured read-range patterns are influenced by the relative polarization alignment between the reader antenna and the tag antenna. Maximum read range is achieved when the polarization of the reader antenna aligns with the dominant current direction of the tag antenna, whereas polarization mismatch results in reduced received power and shorter read range.

The proposed tag antenna was specifically designed for metal-mounted applications; therefore, its performance is strongly influenced by the presence of the metallic backing. With a 200 × 200 mm metal plate, the antenna achieves a peak realized gain of − 6.71 dBi at 919 MHz, which enables a maximum read range of 7.9 m in boresight direction and 10 m in off-boresight angles under 4 W EIRP. In contrast, when the same tag is evaluated in free space without the metal plate, the realized gain drops significantly to − 11.78 dBi, accompanied by a strong impedance mismatch that limits the operational bandwidth and reduces the achievable read range to less than 4.5 m. This behavior confirms that the folded-patch architecture is optimized for metal-backed operation, where the ground plane stabilizes the resonance and enhances radiation efficiency, while in free space the absence of the metallic reflector degrades both gain and read performance.

## Discussion

**Fig. 26:**
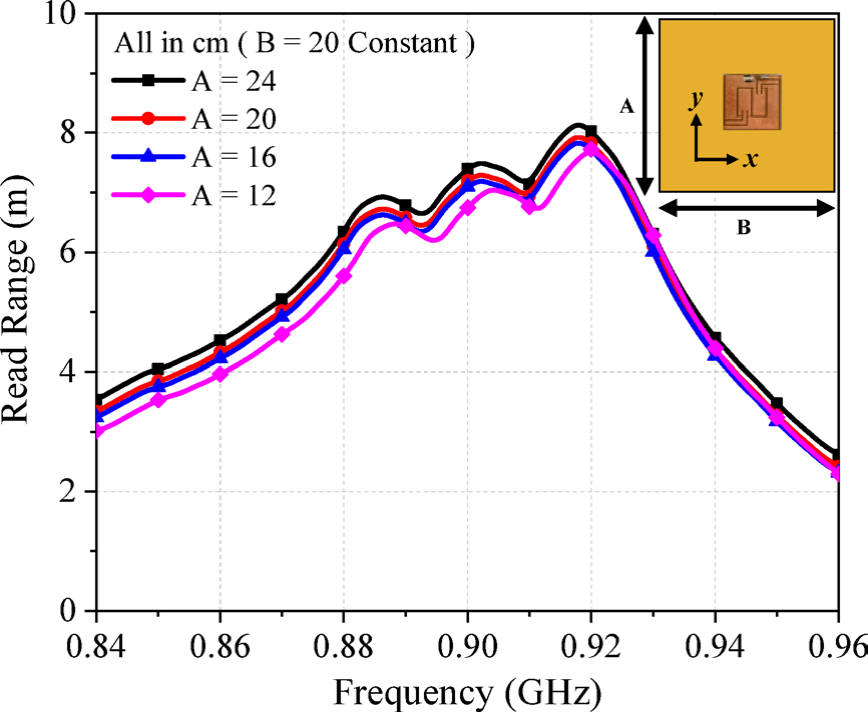
Effect of varying the metal plate length (**A**) on the measured read range of the proposed RFID tag antenna.

**Fig. 27:**
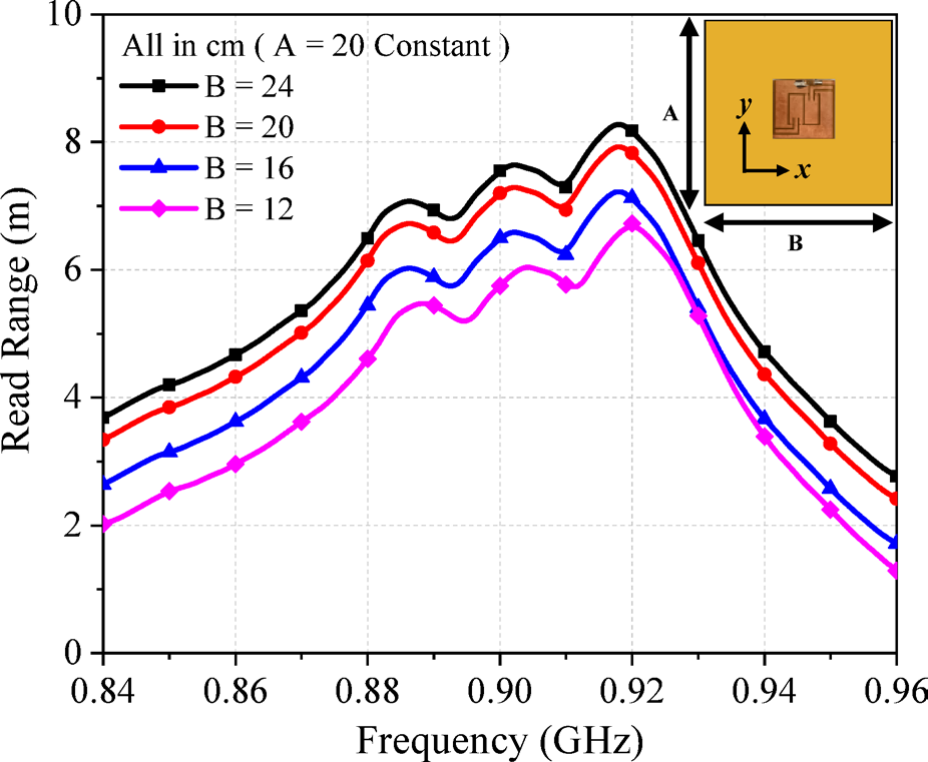
Effect of varying the metal plate width (**B**) on the measured read range of the proposed RFID tag antenna.

The tag was specifically designed for on metal applications and underwent testing on metal plates with varying dimensions, denoted as A × B. The aluminum plates used in the experiments had a uniform thickness of approximately 5 mm, ensuring consistency across the samples. For each test, the tag was consistently positioned at the center of the aluminum plate to standardize the measurement conditions. The read range, a critical parameter for evaluating antenna performance, was measured in the boresight direction (*θ* = 0°). In the measurements, the tag antenna was placed directly on the metallic surface. Since the antenna incorporates its own ground plane, electrical conduction between the antenna ground and the metal surface is not required, and the tag can be mounted using standard non-conductive double-sided adhesive tape for on-metal tag deployment. In the first set of experiments, the width of the plate (B) was fixed at 20 cm, while the length of the plate (A) was gradually reduced from 24 cm to 12 cm. Throughout this process, the read range remained relatively stable, varying slightly between 7.7 m and 8.1 m, as depicted in Fig. 26. This consistent performance highlights the antenna’s robustness and adaptability to changes in plate length within the tested range. In the second set of experiments, the length of the plate (A) was fixed at 20 cm, while the width of the plate (B) was varied. Unlike the first scenario, this adjustment led to a noticeable decrease in the read range, as shown in Fig. 27. This observation suggests that the width of the metallic surface plays a more significant role in influencing the read range compared to its length. Despite these variations in the read range, an important finding was that the resonant frequency of the tag antenna remained stable across all measurements. This stability indicates that the antenna’s frequency response is not significantly affected by changes in the dimensions of the metallic surface, which is a desirable characteristic for consistent performance in practical applications.

**Fig. 28:**
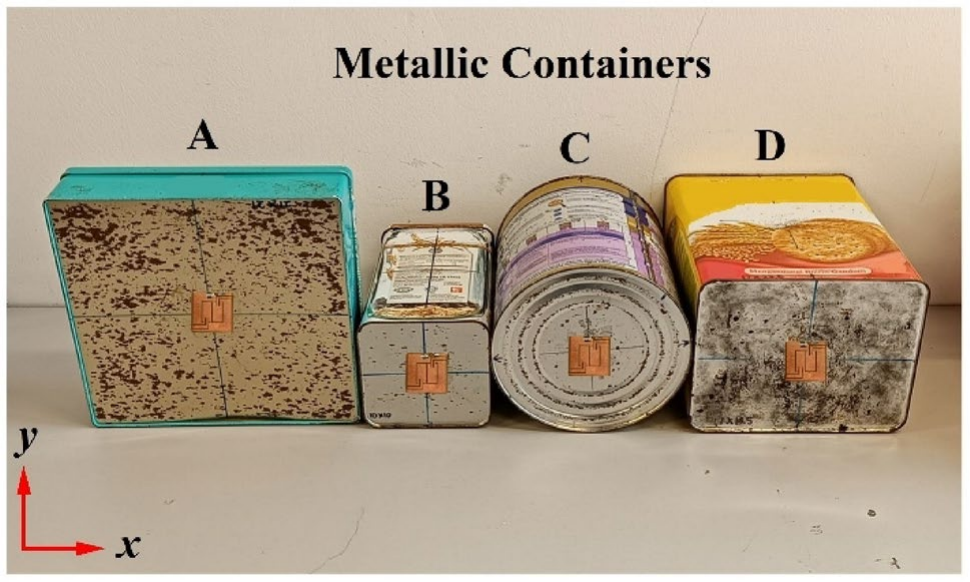
Example of metallic household containers with the proposed RFID tags attached.

**Fig. 29:**
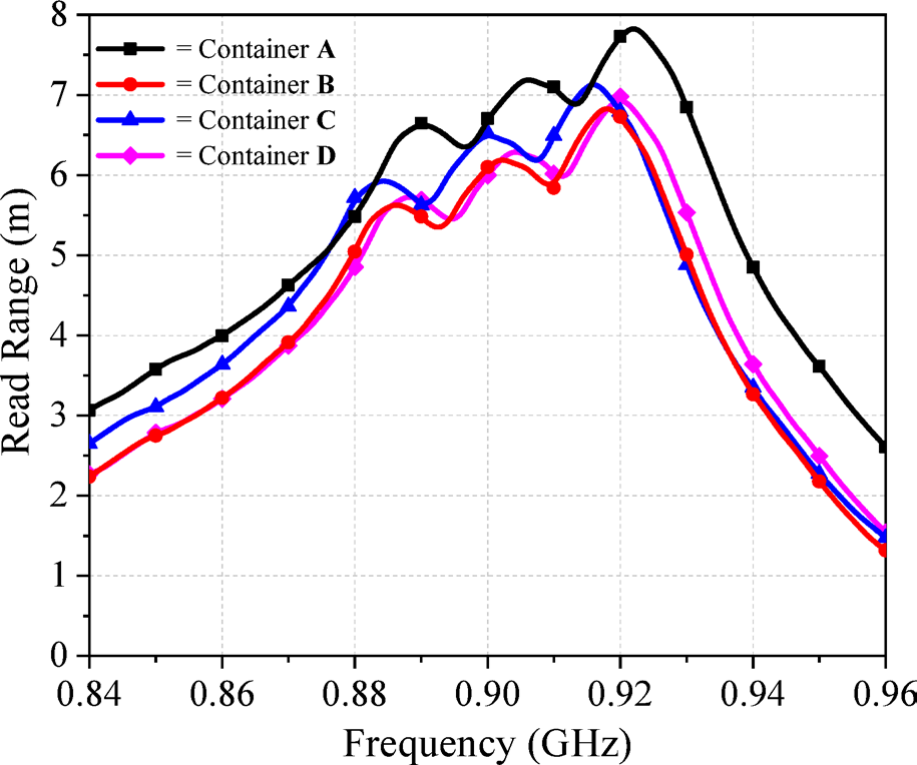
Measured read ranges of the proposed RFID tag antenna for different household metallic containers.

**Fig. 30:**
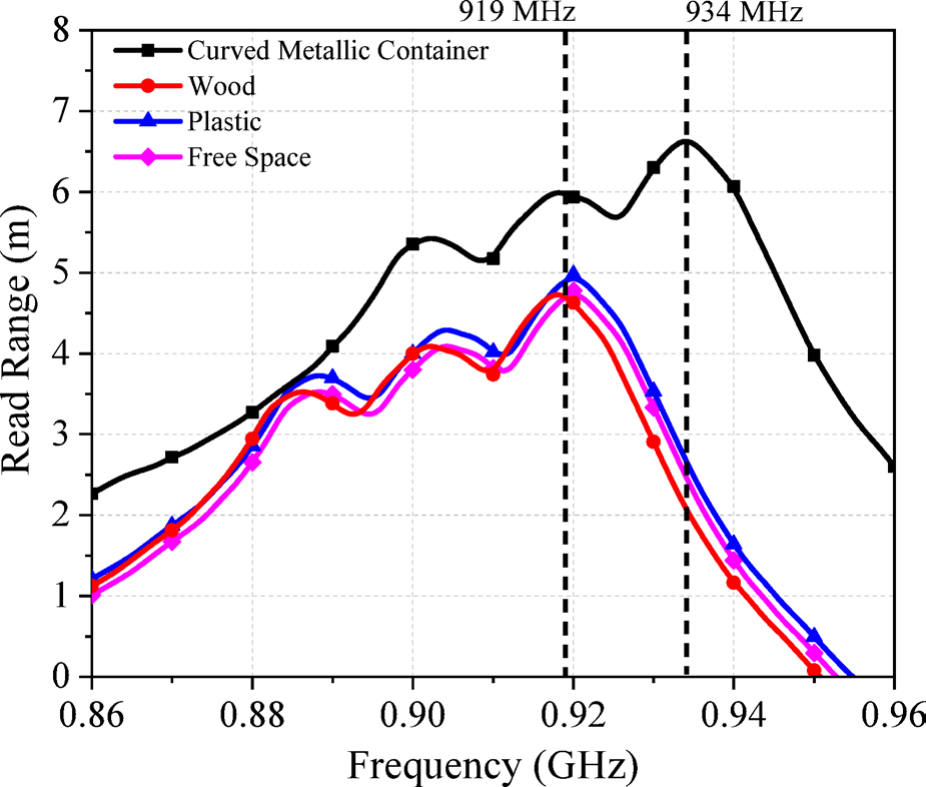
Measured read ranges of the proposed RFID tag antenna on slightly curved metallic surfaces and non-metallic surfaces.

To assess the tag’s performance in real-world scenarios, it was attached to metallic containers of various household items. In these tests, the tag was positioned at the base of each metallic container, as depicted in Fig. 28, and the read range was measured in the boresight direction (*θ* = 0°). As shown in Fig. 29, the tag achieved a read range of approximately 7 to 8 m when attached to household items. A reduction in the surface area width-wise led to a decrease in the read range, primarily due to the use of a linearly polarized reader antenna for measurement. Maintaining the tag’s resonant frequency from 916 to 921 MHz ensured consistent functioning, making the tag antenna highly suitable for practical applications. Figure 30 presents the measured read ranges of the proposed tag antenna when mounted on different objects, including a slightly curved metallic container, wood, plastic, and free space. The tag achieves a maximum read range of approximately 6.5 m on the slightly curved metallic container, while on wood, plastic, and free space, the read ranges remain nearly identical approximately 4.5–5 m. This similarity is expected, as non-metallic materials are not perfect electrical conductors and therefore behave similarly to free-space mounting. A slight resonance shift of about 15 MHz is observed on the curved metallic container, which is normal and occurs due to the curvature modifying the current distribution and boundary conditions. It should also be noted that the flexibility of the proposed tag is intended for uneven metallic objects. Excessive bending is not recommended, since it may break the chip bonding and collapse the folded structure, leading to degraded performance. Thus, the proposed tag demonstrates stable adaptability across both metallic and non-metallic surfaces, with flexibility limited to mild curvatures. The small fluctuations observed in the measured read range curves are mainly attributed to practical measurement conditions, including threshold power sweep variations, calibration uncertainties of the RFID measurement system, and minor multipath reflections within the measurement environment. In addition, the use of the packaged UCODE-9 chip introduces parasitic impedance compared with the bare-die impedance used in simulations, which may slightly alter the effective matching at certain frequencies. These factors may introduce minor fluctuations in the measured read range; nevertheless, the overall trend remains consistent with the simulated antenna gain and impedance matching characteristics.

**Table 2:** A comparative analysis with other tags (Power = 4 W - EIRP).

References	Tag dimension (mm^3^)	Max. realized gain (dBi)	Chip sensitivity (dBm)	Frequency (MHz)	Design complexity (based on number of layers, radiator design, use of vias, and lumped components)	Half power bandwidth (τ ≥ 0.5) (MHz)	Read range (meters)
^26^	50.0 × 50.0 × 3.38	−4.12	−20.85	915	Complex - multilayer Structure	3	9.0
^27^	40.0 × 40.0 × 3.32	−2.90	−20.85	915	Complex - highly sensitive dual rings	16	15.0
^39^	40.0 × 40.0 × 3.32	−0.23	−20.85	915	Simple - no vias & slot loaded design	4	16.0
^40^	47.0 × 21.0 × 2.36	−8.01	−20.85	920	Complex - multilayer structure	76	7.0
^41^	40.0 × 5.00 × 3.20	−3.00	−20.00	915	Complex - multilayer structure	8	11.0
^42^	50.0 × 50.0 × 3.38	−1.90	−20.85	868	Complex - multilayer with lumped inductor	8	14.0
^43^	32.0 × 40.0 × 3.35	−0.41	−20.85	918	Complex - multilayer structure	14	16.0
^44^	40.0 × 40.0 × 1.60	−7.04	−19.90	914	Complex − 2 port chip with 4 shorting stubs	8	7.7
^45^	35.0 × 35.0 × 3.20	−5.41	−20.85	910	Complex - sensitive shorting via	21	9.2
^46^	50.0 × 50.0 × 1.10	−5.70	−18.60	915	Simple - slot loaded design without vias	12	7.3
^47^	20.0 × 20.0 × 3.32	−2.10	−23.00	912	Complex - multilayer structure	10	16.0
^48^	40.0 × 38.0 × 1.57	−5.72	−21.85	920	Simple - Single Layer without Shorting Stubs	60	9.0
^49^	50.0 × 40.0 × 3.32	−1.21	−21.85	918	Simple - single layer without shorting stubs	15	14.0
^50^	53.5 × 12.0 × 3.30	3.00	−20.50	915	Simple – single layer without vias	462 (10 dB BW)	10.2
**This work**	**20.0 × 20.0 × 3.32**	**−6.71**	**−21.85**	**919**	**Simple – Single layer**,** no vias and slot loaded**	**42**	**7.9**

The operational bandwidth (855–942 MHz) of the proposed design is defined based on t he read-range criterion (*R* ≥ 4 m), which reflects practical RFID performance. In contrast, the half-power bandwidth listed in this table corresponds to the power transmission coefficient (PTC) with (τ ≥ 0.5). These two bandwidth definitions are fundamentally different and should not be directly compared.

A detailed comparison of the proposed tag with recently reported on-metal RFID tag antennas is presented in Table 2, considering key parameters such as antenna dimensions, realized gain, chip sensitivity, half-power bandwidth, read range, and overall design complexity. In this table, the half-power bandwidth is derived from the power transmission coefficient (PTC). The double-layered serpentine antenna^26^ was designed for UHF RFID metal applications but suffers from large size and complex dual-layer geometry, which makes it unsuitable for mass production and challenging for compact objects. Additionally, the close proximity of two serpentine patches complicates the design process. In^27^, a compact on-metal tag antenna based on two concentric step-impedance rings was introduced, where the concentric resonators were employed to achieve strong coupling with the chip and extend the operational bandwidth. A similar strategy was later adopted in^39^, where a non-resonating patch was integrated with a planar inverted antenna structure to realize a compact tag for metallic platforms. Both designs demonstrated excellent impedance matching characteristics and achieved relatively long read ranges under standard reader conditions. However, the overall footprints of these antennas are almost twice that of our proposed design, making them less attractive for applications requiring compactness and integration on small metallic objects. The larger physical size not only restricts their deployment on space-limited assets but also increases material usage and fabrication cost compared to the more area-efficient configuration proposed in this work.

A double-layer tag antenna (47 mm × 21 mm × 2.36 mm) is developed for broadband on-metal applications^40^. However, the design is complex due to the use of two different substrates and lacks significant flexibility because of the FR4 substrate. Furthermore, a slim tag antenna with a compact footprint of 40 mm × 5 mm × 3.2 mm, designed for metallic tools, is presented in^41^. The reading range is good, and the tag has slight flexibility due to the foam substrate. However, the three-layer structure is longer in length but very narrow in width, which may cause the tag to bend easily, making it challenging to maintain stable performance. A dipole tag antenna with a top-loaded inductive channel, enabling broad frequency tuning capability, and a physical size of 50 mm × 50 mm × 3.38 mm, was proposed in^42^. In^43^, a folded-patch tag antenna employing a double-layered configuration with multiple inclined slots was proposed for on-metal applications. The inclined slots improve impedance matching and contribute to a higher read range compared to conventional compact structures. However, the use of two stacked substrates together with multiple slot features significantly increases fabrication complexity and alignment requirements. In addition, the larger overall dimensions of this design limit its suitability for integration into smaller metallic assets and increase material usage. While such antennas demonstrate strong read performance, their complexity and size make them less practical for cost-effective mass production, especially when compared to the simpler, single-layer folded-patch structure proposed in this work.

In^44^, a folded crossed-dipole structure was investigated for designing a compact metal tag; however, it exhibited poor read range. In^45^, a novel low-profile top-loaded monopole antenna was proposed for on-metal applications. This design utilizes a via in the middle to short the top radiator to the ground, making it highly complex to design and increasing manufacturing costs for mass production. Additionally, implementing vias in the foam substrate presents significant challenges, further complicating fabrication. The bendable folded patch antenna is proposed in^46^. However, the tag size is nearly double that of our proposed design and offers a lower read range. Conversely, the newly proposed tag antenna provides several distinct advantages. The folded dipolar patch antenna presented in^47^ offers impressive tuning range and read performance but employs a more complex multilayer structure involving a capacitive middle patch and diagonally positioned shorting stubs. The single-layer symmetric dipole planar tag antenna in^48^ offers a wide tuning range for on-metal applications. However, despite achieving a comparable read range, its footprint is nearly twice that of the proposed design. A capacitively coupled meandered-slot serrated patch tag antenna for metal-mountable applications is presented in^49^. While it achieves a slightly higher read range, its size is more than double that of the proposed compact design. The tag in^50^ achieves higher reading range and bandwidth but requires a significantly larger footprint. Designs using extended current paths or serrated geometries are also more frequency-sensitive, where small dimensional variations impact impedance and radiation performance. Additionally, larger structures are more prone to mechanical deformation in practical scenarios. In contrast, the proposed compact design offers improved robustness and stable performance, making it more suitable for space-constrained metallic environments. Additionally, in the comparison table, the design complexity is qualitatively categorized based on the antenna structural configuration and fabrication requirements. Antennas employing single-layer structures with relatively simple geometries are classified as Simple, whereas designs incorporating multi-layer configurations, multiple loading elements, or more intricate geometrical features are categorized as Complex.

In contrast, the proposed tag achieves comparable performance through a single-layer folded-patch configuration with symmetrically embedded open stubs. These stubs extend the effective current path while introducing controlled capacitive coupling, enabling wideband impedance matching without the serrations, multilayer stacking, or loop coupling seen in earlier works. The single-layer architecture eliminates the need for interlayer alignment and metallic vias, simplifying fabrication, reducing cost, and improving reliability. With a footprint of only 20 × 20 × 3.32 mm³, the tag is approximately 36% smaller than many compact counterparts, making it well-suited for metallic objects with limited surface area. Despite its reduced size, the antenna demonstrates robust read performance, achieving a minimum of 7.9 m at *θ* = 0° and a maximum of 10 m at *θ* = ±30° in the *xz* and *yz* plane boresight directions, while maintaining a maximum read range of 8 m in the *xy* plane. Furthermore, the antenna achieves an operational bandwidth of 855–942 MHz, defined based on the read-range criterion, where the tag maintains a minimum read range of 4 m (*R* ≥ 4 m) across the operating band. It is important to distinguish between two bandwidth definitions used in this work. The operational bandwidth is defined based on a practical read-range criterion, where the tag maintains a minimum read range of 4 m (*R* ≥ 4 m). This metric reflects real-world RFID performance and depends on the combined effects of antenna gain, impedance matching, and chip sensitivity. In contrast, the half-power bandwidth is defined using the power transmission coefficient (PTC), corresponding to *τ* ≥ 0.5, which represents the conventional impedance matching bandwidth. Since the read-range criterion does not require peak power transfer, the operational bandwidth can exceed the half-power PTC bandwidth. Since the read-range criterion does not require peak power transfer, the operational bandwidth can exceed the half-power PTC bandwidth. These results highlight the novelty of the proposed design as a compact, manufacturable, and broadband tag with stable performance on metallic surfaces.

## Conclusion

The proposed work presents a simple and compact dual open-stub loaded on-metal UHF RFID tag antenna resonating at 919 MHz. The antenna achieves an operational bandwidth of 855–942 MHz defined using read range *R* ≥ 4 m criterion, where the tag maintains a minimum read range of 4 m across the operating band. A single-layer folded patch antenna consists of two open stubs symmetrically placed to generate high inductance, facilitating frequency tuning of the antenna and achieving conjugate matching with the microchip, thereby enhancing power transfer. The resonant frequency required is obtained by adjusting the width of both open stubs. An in-depth analysis was carried out to gain a comprehensive understanding of the resonance characteristics, and the equivalent circuit model was also developed to enable the precise design of the proposed tag antenna. Experimental measurements were performed to verify the simulation results, revealing a maximum reading range of approximately 7.9 m at boresight, when the tag was placed at the center of a 20 cm × 20 cm metal plate and the proposed antenna is evaluated under direct conductive contact with the metallic surface to ensure a stable ground reference; the effect of an insulating layer will be investigated in future work. The small discrepancies between simulated and measured results are mainly attributed to practical factors such as the difference between the bare-die chip model used in simulation and the packaged RFID chip used in the fabricated prototype, as well as fabrication and measurement tolerances.

## Data Availability

The datasets used and/or analyzed during the current study are available from the corresponding author upon reasonable request.
